# Common mental disorders in Gestalt therapy treatment: a multiple case study comparing patients with moderate and low integrated personality structures

**DOI:** 10.3389/fpsyg.2023.1304726

**Published:** 2023-12-20

**Authors:** Raphaela E. Kaisler, Manfred Fede, Ulla Diltsch, Thomas Probst, Yvonne Schaffler

**Affiliations:** ^1^Department for Psychosomatic Medicine and Psychotherapy, University for Continuing Education Krems, Krems, Austria; ^2^Department Psychotherapy, Bertha von Suttner Private University St. Pölten, St. Pölten, Austria; ^3^Integrative Gestalttherapie, Österreichischer Arbeitskreis für Gruppentherapie und Gruppendynamik, Vienna, Austria; ^4^Division of Psychotherapy, Department of Psychology, Paris Lodron University Salzburg, Salzburg, Austria

**Keywords:** Gestalt therapy, common mental health disorders, operationalized psychodynamic diagnostic, mixed-methods case study, outcome, process, psychotherapy

## Abstract

**Introduction:**

Empowerment is central to self-development and growth in Gestalt therapy. The self evolves through interactions with others, forming self- and object-relations, and ego-functions. Underlying structural functions build the ability to regulate, differentiate, and integrate experiences, leading to self-, and emotion-regulation. Our study examined the self-development of seven clients with prevalent mental health issues and structural challenges, all of whom underwent 30 sessions of Gestalt therapy in a real-world individual therapy context.

**Methods:**

Using a multiple case study approach, we contrasted two client groups: those with moderately integrated and those with low-integrated personality structures, as defined by the operationalised psychodynamic diagnostic manual. Our exploration centered on specific factors of empowerment, therapy processes, and interventions. The study's mixed-method design encompassed quantitative outcome measures (empowerment, wellbeing, psychosocial health, and severity of personality functioning), therapy diaries from both clients and therapists, and semi-structured client interviews about empowering factors in therapy.

**Results:**

Both groups showed positive therapy outcomes on wellbeing, psychosocial health, and empowerment. Specific empowerment-related factors included promoting experiences, relationships, and self-efficacy in the low-integrated group. Support of self-regulation was reported to be essential for successful outcomes in the moderately integrated group. While the therapy processes proceeded similarly in both groups, we observed a strong focus on body awareness-oriented interventions and promotion of verbalisation in the low-integrated group and a relationship-oriented emphasis in the moderately integrated group. Emotional experience linked to positive experience was limited in the low-integrated group, suggesting an impairment of emotional processing, including bodily felt feelings. No change was reported in the level of personality functioning after 30 sessions in both groups.

**Discussion:**

These results underscore the need for tailored therapeutic approaches based on the client's level of personality integration. Future research should probe the long-term effects of therapy and delve deeper into shifts in personality functioning, especially concerning emotional and bodily experiences. In practical terms, therapists should prioritize linking bodily sensations with emotions for clients with low-integrated personalities. For those with moderate integration, the emphasis should be on fostering exploration, awareness, and bolstering self-regulation.

## 1 Introduction

Depression is the most prevalent mental disorder. It often appears alongside anxiety, affecting up to half of those with anxiety disorders (Kroenke et al., 2010). Whilst common, conditions such as depression or anxiety carry significant implications both for the affected individuals and for society at large. These include long-lasting effects and substantial societal expenses (Kroenke and Unutzer, 2017). Research on Gestalt therapy has demonstrated its effectiveness in treating various clinical disorders, including depression and anxiety (Schigl, 1998; Bargghaan et al., 2002; Harfst et al., 2003; Elliott et al., 2004, 2021; Struempfel, 2006a,b; Hartmann-Kottek, 2014). Rooted in the humanistic psychotherapy tradition, Gestalt therapy adopts a holistic perspective of individuals. It sees them as integrated entities whose physical, mental, and cognitive components are closely tied to their social and environmental contexts. This framework encourages therapists to focus on process- and emotion-focused interventions (Elliott et al., 2021). Relevant experience-oriented interventions in Gestalt therapy include exercises to enhance and differentiate body awareness—a process that involves guiding clients to become more attuned to and able to interpret their bodily sensations, recognizing how these sensations connect to particular emotional states and behaviors. Alongside this, interventions also encompass imagination, visualization, and relationship work, each of which plays a crucial role in the therapeutic journey (Struempfel, 2006b). Beyond these, therapists delve into dreams, metaphors, body images, and emotion- and experience-activating Gestalt dialogues (Greenberg et al., 2003; Greenberg, 2015). Gestalt therapy aims to catalyse personal growth and fortify a sense of empowerment in clients. This approach's efficacy is underscored by research, which indicates that treatments centered on empowerment can notably elevate self-esteem and the overall sense of empowerment post-treatment (Lecomte et al., 1999; Stevenson et al., 2003; Borras et al., 2009).

Empowerment in the context of social psychiatry is defined as the promotion of self-initiative (Prins, 2007) to improve people's ability to shape their social environment and their lives (Stevenson et al., 2003). It fosters a client's sense of autonomy and agency, enabling them to recognize and utilize their internal resources for decision-making and coping with life's challenges (Knuf, 2004). Empowerment is also closely linked to self-awareness, a cornerstone of Gestalt therapy; as clients become more aware of their thoughts, emotions, and behaviors, they gain the power to change them (Perls et al., 2015). It is often used in clinical settings (Kliche and Kroeger, 2008; Whitley and Drake, 2010) as part of a multidimensional concept of recovery that positively impacts people with chronic mental illness (Corrigan et al., 1999; Hansson and Bjorkman, 2005; Lloyd et al., 2010). It is positively associated with recovery (Stuart et al., 2017) and, therefore, is recommended for the psychosocial treatment of people with severe mental disorders (DGPPN, 2019). Structural functioning is defined as “the availability of psychic functions necessary for the organization of the self and its relationships with internal and external objects” (Rudolf, 2013, p. 54). It is integral to the process of empowerment in psychotherapy. It influences an individual's ability to regulate emotions, gain self-awareness, cope with challenges, and ultimately engage in a self-directed process of growth and change (Rudolf, 2013; OPD, 2014). Understanding and working with an individual's structural functioning can guide the therapeutic process toward fostering a more empowered and integrated self (Hochgerner and Schwarzmann, 2018). However, the specific treatment pathways leading to successful Gestalt therapy outcomes in patients with moderate or low levels of structural functioning integration remain unexplored.

**Figure 1:**
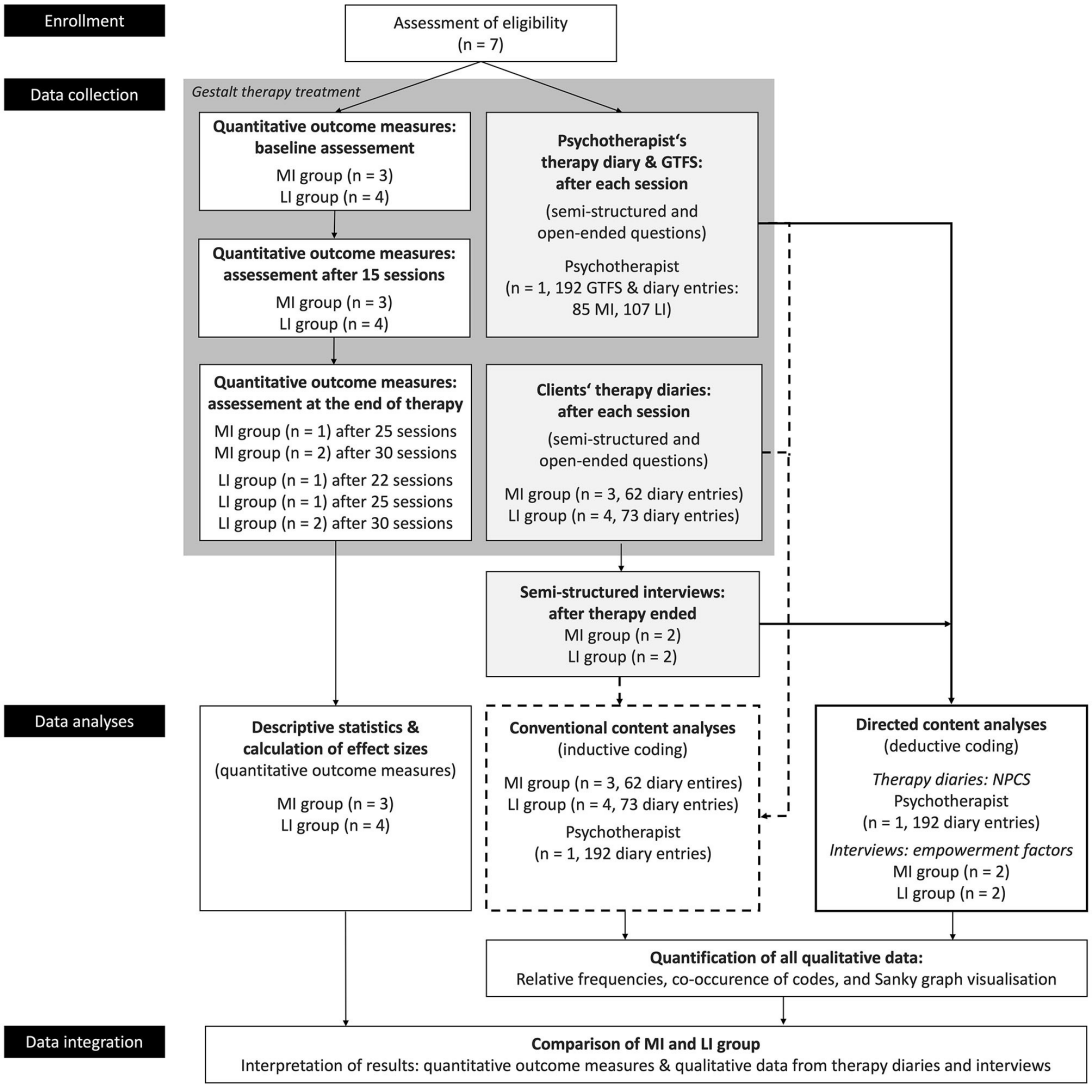
Overview of the study design. The graph depicts data collection in Gestalt therapy treatment using a parallel research design: quantitative data (white boxes) from outcome measures and qualitative data (light gray boxes) from therapy diaries. The therapist also self-reported on the Gestalt therapy fidelity scale (GTFS) after each session. Sequentially, post-treatment, semi-structured interviews were conducted. Thick lines represent directed content analysis, and dotted lines represent conventional content analysis. Integration of quantitative and qualitative data occurred during the comparison of MI and LI groups.

Therefore, in this study, we explored the empowerment concept as a primary outcome for treating common mental health disorders with Gestalt therapy. We aimed to understand the therapy process and how structural functioning plays a part in the treatment. We examined various cases focusing on clients with moderately integrated (MI) or low-integrated (LI) structures. After 30 therapy sessions, both MI and LI groups showed positive results. However, the therapeutic journey to these results varied between the groups. To pinpoint the specific processes, techniques, and factors that led to empowered clients, we gathered insights from the therapy diaries of both clients and therapists. We also interviewed clients after their treatment concluded (a summary of our study design can be seen in Figure 1). Our approach was a comparative study across multiple cases to shed light on the therapy processes and what they mean for practical application. Before diving into the specifics of our research design, it is essential to clarify the ideas of empowerment and self-growth. We base our explanations on the foundational principles of Gestalt therapy and the significance of structural functioning.

### 1.1 Gestalt therapy

Gestalt therapy is a form of psychotherapy developed by Fritz and Laura Perls, Ralph Hefferline, and Paul Goodman in the 1940s and 1950s. It is rooted in existential philosophy, influenced by Gestalt psychology, and focuses on the holistic perception of experience. The Gestalt approach emphasizes the individual's experience in the present moment and the importance of the therapist–client relationship. A central concept is the “figure-ground” distinction, which helps individuals organize their perception by differentiating between what is at the forefront of their attention and what is in the background. Addressing unresolved issues or “unfinished business” is essential in Gestalt therapy, providing individuals with a sense of closure and emotional freedom. The therapy also promotes enhanced self-awareness and a deeper understanding of one's interactions with others. It champions the idea that genuine contact with oneself and the surrounding environment is a cornerstone for personal growth. Moreover, embracing one's current state is believed to lead to authentic transformation. To facilitate this understanding, Gestalt therapy employs various experiential techniques. For instance, the “empty chair” technique allows clients to converse with an imagined person or aspect of themselves. Other techniques, such as re-enacting dreams, provide a means to explore feelings and experiences, while role-playing can be used to confront and address internal conflicts (Perls et al., 2015).

The Gestalt session is not rigidly structured. Instead, it is more fluid and organic, focusing on the present moment and the unfolding process of the client's experience. However, certain elements and principles underpin the session's progression: establishing the therapeutic relationship, a present-centered focus, and raising awareness of emotions, thoughts, and behavior. Gestalt therapists suggest experiments and exercises so the client can engage with new experiences, work with resistance, and create a dialogic environment by providing feedback and shared observations. Closure toward the end of sessions helps to reflect on insights, and sometimes, the therapist suggests “homework” between sessions, fostering reflections.

In Austria, Integrative Gestalt therapy (as applied in this study for treatment) is a recognized psychotherapy approach for the treatment of mental illness. Among the described vital concepts and experience-oriented working, it incorporates developmental psychological and psychodynamic perspectives to address self-development from childhood to maturity (Klampfl and Hochgerner, 2022). Here, we outline the process of self-development and the implications of structural problems in treatment.

### 1.2 The self and structural development

Gestalt therapy perceives the self as a constantly evolving “system of contact at any moment” that seeks to realize its potential for growth (Perls et al., 2015, p. 31). Modern perspectives (Staemmler, 2018) depict the self as a fluid entity characterized by its dynamic nature and multiple facets (Polster and Polster, 2003; Perls et al., 2015). The formation of the self depends heavily on interactions with others (Staemmler, 2015; Boeckh, 2019a). From early in life, there is a dialogical relationship with others (Stern, 1985, 2011), a concept supported by Buber (2017). Essentially, our understanding and experience of the self are rooted in our interactions with people and our environment, underscoring the deep bond between the self and its surroundings (Spagnuolo-Lobb, 2016b).

From a phenomenological view, the self is an embodied consciousness encompassing an individual's biological and psychological aspects (Merleau-Ponty, 1966; Petzold, 1993). The self evolves through our interactions with our surroundings. These interactions help develop essential ego-functions. For instance, basic functions (primary ego-functions), such as perception and emotion, lay the groundwork for more complex abilities (secondary ego-functions), such as impulse control, introspection, and empathy (Klampfl and Hochgerner, 2022). The self is not static; it continually evolves, forming dynamic and procedural (physical) structures (Damasio, 2011; Votsmeier-Röhr, 2011). L. Perls (Sreckovic, 2005) suggests that these structures build the “support” in the background for the processes in the foreground. Wheeler (2000) emphasizes the importance of being fully present, building meaningful connections, and delving into our internal world of self-development. However, our growth can be stunted. Factors such as deficit, trauma, malfunction, stress, and conflict can obstruct our self-development (Petzold, 1993). This often results in symptoms of rigid and dysfunctional behavior (Perls et al., 2015; Klampfl and Hochgerner, 2022).

L. Perls (Sreckovic, 2005) underscores the role of self-support, especially when addressing early life disturbances. She believes that a strong sense of self-support is essential for meaningful interactions and the overall growth of the self. Taking a broader perspective, the Gestalt theory of self, as proposed by Spagnuolo-Lobb (2012), presents the “Polyphonic Development of Domains.” This concept posits that various areas of our lives, be they personal, relational, or cultural, evolve side by side, each influencing and shaping the other.

The psychodynamic perspective similarly suggests that our sense of self and ego-functions take root in childhood and are shaped by our interactions with others (Rudolf, 2000). Our memories, both from real-life events and our inner reflections, play a crucial role in this development (Stern, 1985, 2011). These memories influence how we perceive ourselves and others and are closely tied to specific effects (Kernberg, 1981; Bacal et al., 1994; Sandler and Sandler, 1999). At the core of this development are structural functions that help us regulate, distinguish, and merge our experiences (Rudolf, 2002). As the self matures, it achieves a sense of existence and the ability to form meaningful connections with others. This maturity manifests in several ways: the ability to articulate emotions, a heightened awareness of one's physical self, and a clear distinction between oneself and others (Rudolf, 2013). This growth ultimately leads to a well-defined sense of identity (OPD, 2014). A vital feature of this mature self is its reflective nature. It draws from internalized perceptions and forms a self-image, which is crucial for regulating emotions and the self (Fonagy et al., 2002).

Furthermore, within the framework of Gestalt therapy, the mature self is linked to the embodied feelings of existence that originate in early childhood. This intrapersonal sense of the certainty of one's existence, known as “Daseinsgewissheit,” evolves through interpersonal experiences shaped by the emotional and physical responses of caregivers. Positive resonance experiences with others contribute to the formation of emotional, social, and cognitive intrapsychic representations. These early interactions lay the groundwork for the development of self-assurance, termed “Selbstgewissheit.” This self-assurance is built upon primary ego-functions, which, in turn, help form emotional, cognitive, and psychological perceptions of others (Petzold, 1993; Hochgerner and Schwarzmann, 2018).

Deficits in structural functioning can lead to challenges in managing relationships with oneself and others. Additionally, these deficits can impact one's ability to understand and reflect on mental states. As a result, individuals become more vulnerable to overwhelming and traumatic situations throughout their development (Rudolf, 2013). These structural (mal)functions can be classified diagnostically (well, moderate, low, and disintegrated structures) and therapeutically according to the operationalised psychodynamic diagnostic manual (OPD, 2014), which has been cross-validated with structural malfunctions (Rudolf, 2013; OPD, 2014). In recent times, the OPD has become increasingly significant in the realm of Gestalt therapy. Numerous studies (Votsmeier-Röhr, 2013; Hochgerner et al., 2018; Klampfl and Hochgerner, 2022) have integrated psychoanalytical and psychodynamic principles to offer more nuanced diagnoses and treatments. This study utilized the OPD manual to evaluate its clients' personality structure and functioning.

### 1.3 Treatment of structural problems in Gestalt therapy

People with structural challenges often face difficulties in relationships and may feel less capable when under stress. This makes the bond between the therapist and client vital, as it provides a supportive environment for the clients to express themselves (Hochgerner et al., 2018). Therapists need to cultivate specific relational skills to bolster the client's sense of responsibility (Yontef, 1999; Spagnuolo-Lobb, 2016a). This relationship also aids in incorporating bodily experiences, as many of these clients tend to be disconnected from their physical sensations. This desensitization or restriction in bodily sensation (Kepner, 2010) stems from past overstimulation or developmental challenges and manifests as defensive behaviors or coping mechanisms (Petzold, 1993). To enhance emotional control, understanding of mental states, and ego-integration (Votsmeier, 1999; Votsmeier-Röhr, 2005, 2011; Wöller, 2013), therapists often employ L. Perls' contact-support model (Sreckovic, 2005). This model emphasizes the development of contact functions through adequate support. It incorporates creative techniques (e.g., drawing), body awareness, and self-aspect exploration (Hochgerner et al., 2018). These experience-oriented techniques promote perception and stimulate reflection (Hochgerner, 2015). They help internalize relational experiences (Rudolf et al., 2008) and broaden one's ability for self-regulation and self-support (Gremmler-Fuhr, 1999). The key is to recognize and understand current experiences and situations (Yontef, 1999). Empirical evidence from Hochgerner and Schwarzmann (2018) has shown that such experience-based Gestalt therapy approaches are effective for psychosomatic patients with structural deficits.

Emotions take center stage in Gestalt therapy (Boeckh, 2019b). They are pivotal for both emotional processing and structural functioning. For emotions to be processed effectively during therapy, a strong therapeutic alliance is essential (Beutler et al., 2000; Horvath, 2005). Interestingly, heightened emotional states during therapy sessions often indicate positive outcomes, especially when combined with a strong alliance (Iwakabe et al., 2000). Access to and awareness of emotions are key factors that influence the therapy process and outcome (Bohart and Greaves Wade, 2013), and they depend on the patient's pre-treatment emotional processing characteristics (Brintzinger et al., 2021). Emotional processing involves becoming aware of emotions, enhancing emotion regulation, reflecting on emotions, and transforming them (Greenberg and Pascual-Leone, 2006). Yet, this alone is insufficient for lasting change (Greenberg and Pascual-Leone, 2006; Greenberg, 2015). True transformation involves blending thoughts and feelings, understanding emotions, and using language to structure and assimilate emotional experiences (Greenberg, 2002). For more profound, more meaningful change, it is beneficial to concentrate on bodily felt experiences (Gendlin, 1996) and derive new understandings from them (Samoilov and Goldfried, 2000; Greenberg, 2002). Petzold (1993) describes the client's synergy of bodily experience, emotional experience, and cognitive understanding as “vital evidence” (p. 694–695). Through the experience of such a synergy, problems are understood by re-experiencing their origins and placing them in the context of the past and the present. “The total of all elements is more and something different than the sum of the individual components or individual effects” (Petzold, 1977, p. 254–255). These new experiences, including the awareness of inner bodily feelings, need to be organized within a narrative framework (Angus, 2012). Angus et al. (1999) and Hardtke et al. (2002) developed the Narrative Process Coding System (NPCS), which examines the nuances of personal storytelling, deriving meaning, and differentiating emotions during therapy. In this study, we employed the NPCS to analyse (1) the psychotherapist's therapy diaries to characterize the therapy process and interventions and (2) the emotional experiences and making meaning of clients. For more details on this dual-perspective narrative process coding approach, see our methods below.

### 1.4 The present study

In this multiple-case study, based on Yin and Campbell (2018) research design framework, we examined how two levels of structural functioning influence empowerment and self-development in individuals with common mental health disorders undergoing Gestalt therapy treatment. Because empirical evidence considering the structural functioning level in Gestalt therapy is limited (Hochgerner and Schwarzmann, 2018), we conducted a mixed-methods study in a naturalistic psychotherapy setting, focusing on seven cases undergoing Gestalt therapy to evaluate outcomes and understand processes. The participants exhibiting moderate and low-integrated structures were divided into two groups: Group (i), subsequently the MI group, consists of three clients with moderately integrated structures, and Group (ii), subsequently the LI group, includes four clients with low-integrated structures. Our objective was to identify experiential patterns between the groups by addressing the following research questions (RQs): (1) How do individuals of each group perceive their therapy outcome after treatment when analyzing quantitative data collected in therapy diaries of clients and pre-post assessment? (2) How do structural problems influence the therapy process according to the therapy diary of the psychotherapist? (3) How do clients perceive their therapy process according to their therapy diaries? (4) Which specific empowerment factors in Gestalt therapy foster self-development when analyzing qualitative data collected in interviews?

Ethical approval for the study was obtained by the ethical committee of the University of Continuing Education Krems, Austria (EK GZ 03/2021-2024), following the Declaration of Helsinki. All participants gave electronic written informed consent for participation in the study, which included completing the questionnaires, therapy diary, the option of taking part in a qualitative interview, and consent to these case details being published.

## 2 Methods

We utilized a parallel (two types of data: qualitative and quantitative) and sequential (pre-, during, and post-therapy) data collection approach (Figure 1): the quantitative data assessed the efficacy of the treatment outcomes. The qualitative data facilitated a deeper exploration of clients' and psychotherapist's perceptions and experiences in the therapy process. Quantitative data were gathered through standardized questionnaires at three time points—baseline, 15, and 30 sessions. Qualitative data were obtained from therapy diaries maintained by both clients and psychotherapists during the treatment phase. Post-therapy, sequential qualitative data were collected through semi-structured interviews.

### 2.1 Clients

Clients were recruited from the Psychotherapeutic Outpatient clinic “Psychotherapeutische Ambulanz (PTA)” in Vienna, Austria, and a private psychotherapy practice in Vienna from September 2021 to December 2022. Seven female clients (*M*_*age*_ = 30.79, *SD* = 9.48) residing in Austria participated in the study, of whom four clients conducted a semi-structured interview after 30 sessions of psychotherapy. International Classification of Diseases 10th Revision (ICD-10) diagnoses encompassed depression, anxiety, posttraumatic stress disorder, and borderline personality disorder (Supplementary Table S1). This study's inclusion criteria were: (a) adults over 18 years, (b) who had currently no other psychotherapy treatment, (c) self-reported common mental health problems existing for a minimum of 6 months, and (d) met the criteria for a common mental disorder. Patients had to have a common mental disorder operationalised as a PHQ-9 (Kroenke and Spitzer, 2002) score of ≥10 and/or a GAD-7 (Kroenke et al., 2007) score of ≥8. These cutoff scores showed adequate sensitivity (GAD-7, 77%; PHQ-9, 88%) and specificity (GAD-7, 82%; PHQ-9, 88%), as reported in Pieh et al. (2021); and (e) they had to have a moderate or low-integrated personality structure assessed by an OPD-2 diagnostic interview (OPD, 2014). The OPD differentiates four levels of structure (well-integrated, moderately integrated, low-integrated, and disintegrated). Moderate integration (MI) implies a lower availability of regulating function and a weaker differentiation of mental substructures than in well-integration. With low integration (LI), the inner mental space and substructures are even less developed. Thus, conflicts are rarely worked out internally but are mainly worked out in the interpersonal sphere. Several empirical studies have shown OPD's predictive, constructive, and clinical validity and reliability (Cierpka et al., 2007).

### 2.2 Treatment

The psychotherapist and certified OPD-2 rater assessed the clients who met the inclusion criteria at the beginning of treatment, according to the OPD diagnostic manual (OPD, 2014). The psychotherapist communicated the study aim—assessing empowerment in Gestalt therapy—and explained the different data collection methods. Furthermore, clients were informed that they could drop out of the study at any time, which would not affect their further treatment. After clients gave written informed consent, the Integrative Gestalt therapy treatment started. In line with the Gestalt approach, sessions were not structured; however, in the first sessions, the psychotherapist also explored symptoms and the clients' biographical background.

The frequency of individual one-to-one Gestalt psychotherapy was once a week. The duration of therapy varied from 22 to 30 sessions of Gestalt therapy. Some clients left the study early because of immediate improvement, and three clients preferred not to continue: one MI client after 25 sessions and two LI clients after 25 and 22 sessions. Four clients (two MI and two LI) received 30 treatment sessions. All clients received treatment from the same psychotherapist in Gestalt therapy. Clients were able to continue with psychotherapy after the study ended. Client demographic data at the onset of the treatment are summarized in the Supplementary Table S1.

### 2.3 Data collection

#### 2.3.1 Quantitative outcome measures

The goal of the quantitative phase was to capture changes in various areas of clients' lives via four self-report psychometric questionnaires (Empowerment Scale, HEALTH-49, WHO-5, SASPD). Clients were instructed to fill in the quantitative outcome measure questionnaires and therapy diary within 3 days after the psychotherapy sessions, starting with the baseline measure questionnaires after the first session. Clients were asked to self-report using the same questionnaires after 15 and 30 psychotherapy sessions for outcome assessment. In addition, the psychotherapist also self-reported each psychotherapy session using the Gestalt Therapy Fidelity Scale (GTFS, Fogarty et al., 2019) to check whether major components of Gestalt therapy were conducted.

We applied the following measures for defining the outcome translated to or validated in German and often used in the research literature to assess mental health and psychological symptoms:

##### 2.3.1.1 Empowerment scale

The questionnaire (Rogers et al., 1997) measures personal empowerment among users of mental health services with 28 self-report items on a 4-point Likert scale on five subscales: self-esteem and self-efficacy, optimism and control over the future, power and powerlessness, activism and autonomy, and righteous anger. The raw scores range from 1 (strongly disagree) to 4 (strongly agree). The English version was validated with an in- and outpatient mental health population (Corrigan et al., 1999; Wowra and McCarter, 1999; Castelein et al., 2008; Barr et al., 2015). Psychometric properties show a relationship to hope, social acceptance, quality of life, and attitudes toward recovery (Rogers et al., 2010). The first author translated it into German for the study, and an experienced researcher checked the translation.

##### 2.3.1.2 HEALTH-49

The Hamburger Modules for the assessment of psychosocial health measure, psychosocial health questionnaire (Rabung et al., 2007), comprises six modules with nine scales, in total 49 self-report items: somatoform complaints, depressiveness, phobic anxiety, psychological wellbeing, interactional problems, self-efficacy, activity and participation, social support, and social stress. The raw scores range from 0 (not at all) to 4 (very much), providing a measure of the intensity and frequency of psychosocial problems on a 5-point Likert scale. These scales have demonstrated high reliability in large clinical and healthy German samples (Rabung et al., 2009).

##### 2.3.1.3 WHO-5

The WHO-5 questionnaire (World Health Organisation. Regional Office for Europe, 1998) measures wellbeing with five self-report items rated on a 6-point Likert scale, with higher scores indicating higher wellbeing. The raw score ranged from 0 (absence) to 25 (maximal) of wellbeing. Afterwards, they were multiplied by four, translating them to a percentage scale from 0 (absent) to 100 (maximal), which indicates the health-related quality of life (Topp et al., 2015).

##### 2.3.1.4 SASPD

The severity of personality disorder was measured with the Standardized Assessment of Severity of Personality Disorder (SASPD, Olajide et al., 2018) questionnaire on nine subscales. The 4-point scale ranges from 0 (absent) to 3 (severe), measuring nine dimensions: being with others, trusting others, friendships, temper, acting on impulse, worrying, being organized, caring about other people, and self-reliance. The SASPD has a good predictive ability for ICD-11 personality disorder criteria. Its retest reliability has been evaluated with clinical and non-clinical German samples (Zimmermann et al., 2019; Rek et al., 2020).

#### 2.3.2 Qualitative process measures

The goal of the qualitative phase was to compare the cases and explain the therapy process in moderate and low-integrated groups. Clients reflected on each session by writing in an electronic therapy diary. They did so by completing closed and open-ended questions about their ongoing therapy experiences. In a complementary fashion, the psychotherapist wrote in an electronic therapy diary to report on the therapy process of each psychotherapy session. After 30 weeks, clients were invited to complete a semi-structured interview (optional) reflecting on empowering factors in their psychotherapy process. Three interviews were held in person and one online via Zoom.

##### 2.3.2.1 Therapy diary

A therapy diary, designed to encapsulate experiential aspects from both the client and the therapist, was bifurcated into two sections: first, a structured questionnaire assessing Gestalt concepts (see Nausner, 2018) with a 5-point Likert scale ranging from 1 (do not agree) to 5 (strongly agree) on six items:

- “I have intensively thought about today's session” (intensity of process),
- “I have succeeded in bringing my request/concern” (self-efficacy),
- “I managed to participate in exercises and experiments” (openness and creativity),
- “I felt bodily reactions today” (body awareness),
- “I felt emotional reactions today” (emotional awareness), and
- “I experienced something special with the psychotherapist or client today” (dialogic relational).

Second, seven open-ended questions addressing the following topics referring to Gestalt therapy (Perls et al., 2015; Nausner, 2018):

- “today's topic,”
- “the following aspects were discussed,” referring to the concept of the whole in Gestalt therapy,
- “my concern/request today was” and “today's session got me thinking about,” referring to the Gestalt forming processes (“figure,” something stands out from the “ground”/context),
- “my meaningful moment today was” or “I didn't have a meaningful moment today” referring to Teschke (2017) “essential moments in therapy,”
- “what else would I like to say about today's session?” referring to the foreground—something that can be fully experienced and coped with,
- and “additional thoughts by the client (e.g., personal goals, concerns, reactions, mood, and complaints)” referring to Butollo (1995) clients' involvement in research documentation. Clients could additionally upload files and pictures to each question.

##### 2.3.2.2 Qualitative semi-structured interview

Two cases from each group (i and ii) agreed to participate in an interview for a more in-depth exploration. Post-treatment semi-structured qualitative interviews with these four clients covered various topics based on the five subscales of the Empowerment Scale (Rogers et al., 1997): self-awareness and self-efficacy, decision-making and autonomy, resources and competencies, social support, and capacity for change and openness. Twelve open-ended questions were used to encourage clients to speak about their experiences and insights gained from the psychotherapy process. For example, the clients were asked to talk about a difficult situation that had occurred since they started therapy and whether they could cope independently. They were also asked how such an experience of self-efficacy felt physically and emotionally. Clients reflected on situations where they had to make decisions and which resources and competencies had supported them since they started therapy. They further talked about situations in which they felt supported by others and whether their interactions with others had changed (Supplementary Table S2). The last author, a qualitative, experienced social scientist, conducted the 1-h-long interviews, which were audio-recorded and transcribed for further data analyses.

### 2.4 Data analysis

#### 2.4.1 Descriptive statistics of quantitative data

We computed pre- (baseline), mid- (15 sessions), and post-treatment (30 sessions) measurement scores for each client and each instrument. Scores were calculated as means (*M*) of the items' responses and standard deviation (*SD*). To compare cases, we summarized scores for the “moderately integrated” (MI) and the “low-integrated” (LI) groups for further analyses and reporting (Supplementary Table S4). Descriptive statistics were conducted using SPSS 27 (IBM Corp, Armonk, NY, USA). As a pre-post effect size measure, Cohen (2013) was calculated by subtracting the pre- or mid- and post-mean values divided by *SD* pre-treatment to categorize small (*d* = 0.2 to 0.5), medium (*d* = 0.5 to 0.8), and large effects (*d* > 0.8).

#### 2.4.2 Content analyses of qualitative data

We conducted qualitative content analyses (Hsieh and Shannon, 2005) with subsequent quantification of qualitative categories (Kyngäs et al., 2019) of the therapy diaries. First, RK read all diaries from the psychotherapist, including a complete entry list of reflections from each client's session. Second, each answer was read word by word to derive inductive codes by paraphrasing quotations to characterize their content. We used the software Atlas.ti (Version 22.2.3) (Friese, 2021), assigning each diary entry of all seven clients (*N* = 135) and the psychotherapist (*N* = 192) to at least one code, thus developing a preliminary list of codes developed through conventional content analysis (Hsieh and Shannon, 2005). In the second step, the larger numbers of codes were subsumed under a smaller number of more abstract categories. RK undertook this allocation. Together with the other two coders (YS and MF), the list of categories was iteratively discussed, adjusted, and organized into a final structure of (sub)categories until a consensus was achieved (Supplementary Table S3).

Third, we additionally applied deductively derived codes (directed content analysis, Hsieh and Shannon, 2005) from the macro narrative framework of the Narrative Process Coding System (NPCS, Angus et al., 1999; Hardtke et al., 2002). NPCS identifies strategies and processes that represent the client's experience of self and others in the world and their making meaning of these situations. It distinguishes between three narrative coding sequences: (a) “external” describes past, present, or future imagined or happened events, (b) “internal” describes elaborations on subjective/experienced emotions and reactions to the events, and (c) “reflexive” refers to reflective analyses of such events that include cognitive (external) and emotional (internal) components. Furthermore, the code “domain shifts” marks if a new theme starts, and “facet shifts” if elaborations on the same theme are explored. The sequences were coded according to their “relationship focus” (self, others, or self in relation to others). Although the model was initially developed for verbal client–therapist interaction during treatment sessions, the authors also applied the NPCS to the psychotherapist's therapy diary, aiming to characterize the therapy processes of the MI and LI groups.

**Figure 2:**
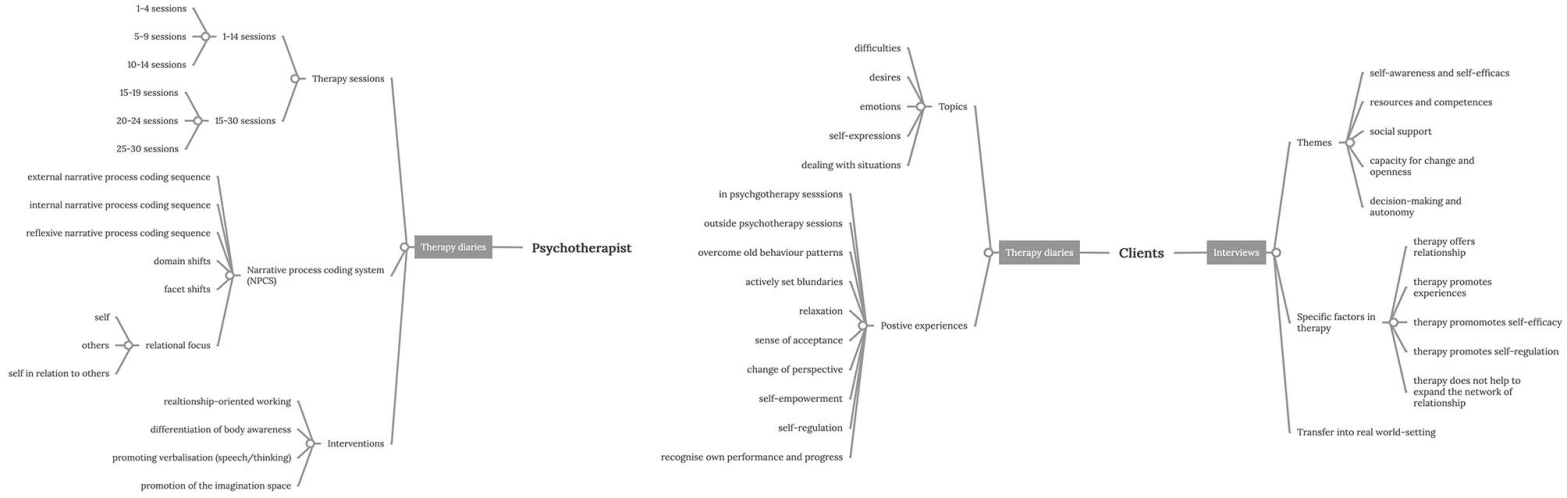
Coding system for qualitative data. The graph displays the coding system of qualitative data sources (psychotherapist's therapy diary, clients' therapy diaries, and interviews) on the level of categories and subcategories (branches). Descriptions of codes are summarized in Supplementary Table S3.

Fourth, we analyzed the four anonymised transcripts from the semi-structured interviews using Atlas.ti (Friese, 2021) as described above. We applied deductively derived codes based on the topics covered in the Empowerment Scale (Rogers et al., 1997) to explore specific factors of empowerment in Gestalt therapy treatment (directed content analysis, Hsieh and Shannon, 2005). Additional subcodes within the topics were derived inductively from the transcripts and were subsumed into categories (Figure 2, Supplementary Table S3).

Fifth, for the quantification of categories, all data points were normalized across cases and code frequencies since both groups differ in their quantity of text. We conducted within- and across-group comparisons (Ayres et al., 2003), calculating absolute and relative frequencies in percent (%). To explore the co-occurrence of codes—codes that have been applied to the same or overlapping quotations—we used the co-occurrence table and Sankey graph visualization in Atlas.ti (Friese, 2021). In our study, co-occurrence indicates that two codes are associated with quotations that refer to the same data segments. We applied this coding system to the therapy diaries of clients, the diaries of the psychotherapist, and the four interviews (Figure 2).

Sixth and last, we compared the qualitative categories with the groups' quantitative data. With this analysis, we could identify (in)congruencies of the different data sources. This step helped us to triangulate the inferences from the qualitative and quantitative data. Using the constant comparison method, we looked for similarities and differences between the MI and LI groups (Boeije, 2002).

## 3 Results

We report our data comparing two groups—the MI group representing three cases and the LI group representing four cases—and present their quantitative treatment outcome that is subsequently compared via the qualitative process analyses. At baseline measures, the LI group displayed ratings above the clinical cutoff score of depression (PHQ-9, *M* = 13.25, *SD* = 2.89) and anxiety (GAD-7, *M* = 16.25, *SD* = 2.99), whereas the MI group did so only in depression (PHQ-9, *M* = 10.33, *SD* = 2.08; GAD-7, *M* =5.67, *SD* = 2.52).

### 3.1 Descriptive statistics of quantitative outcome measures

**Figure 3:**
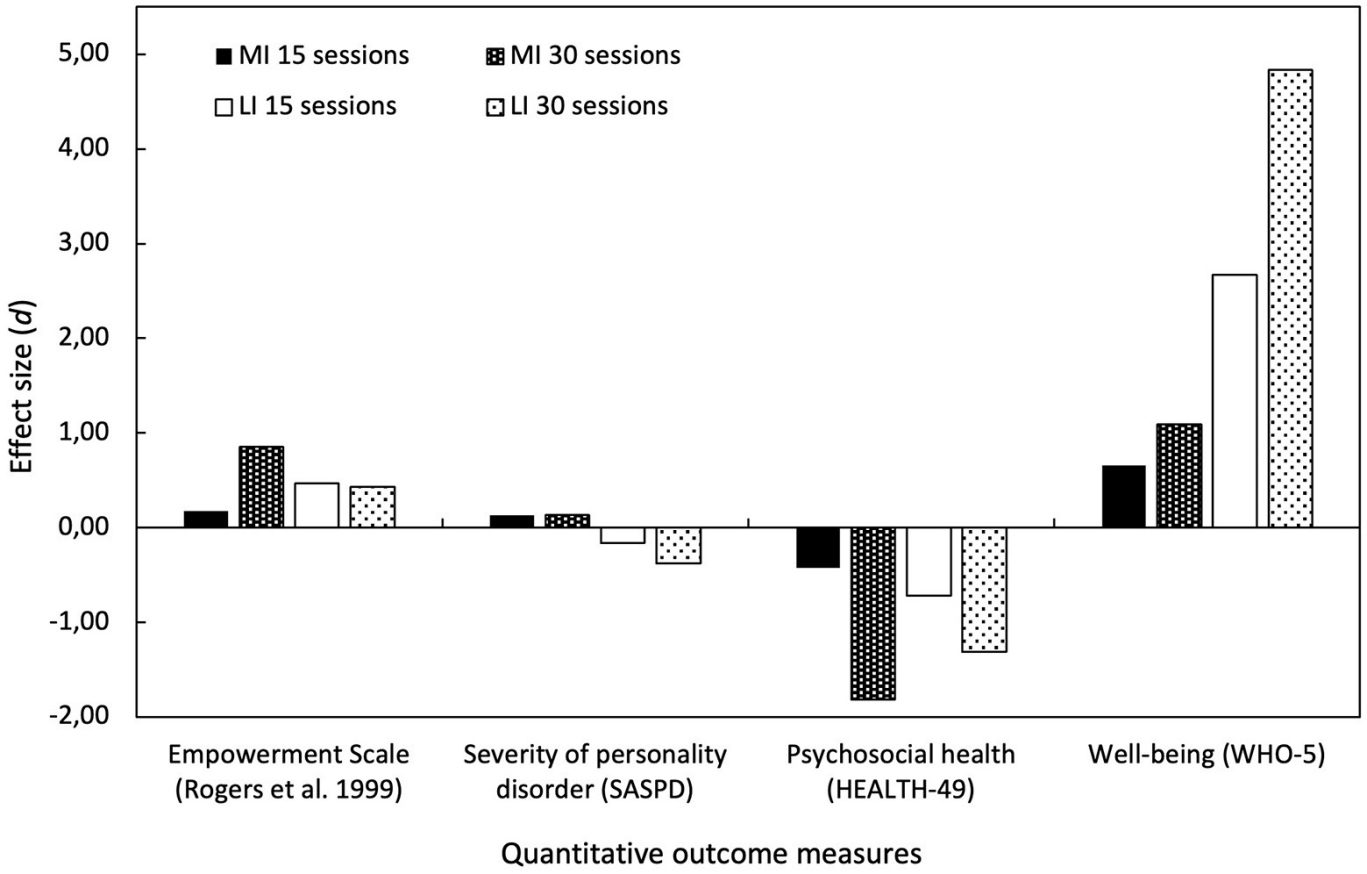
Treatment outcome measures. Overview of pre-post effect sizes from outcome measures after 15 and 30 sessions. Black bars indicate the “moderately integrated” (MI) group and white bars indicate the “low integrated” (LI) group. Solid bars indicate effect sizes after 15 sessions, and dotted bars indicate effect sizes after 30 sessions of psychotherapy compared to baseline measures.

To examine the efficacy of Gestalt therapy treatment for clients with common mental health disorders and a structural problem (RQ1), we first analyzed the treatment outcome of the total sample, both the MI and LI groups, during and after the treatment on the primary and secondary outcome measures. Due to the small sample size, we did not perform statistical tests but calculated pre-post effect sizes for comparisons (Supplementary Table S4, Figure 3). Negative signs of effect sizes indicate a reduction of symptoms, whereas positive signs indicate an improvement compared to baseline. Figure 3 shows an overview of the effect sizes of the Empowerment Scale, SASPD, Health-49, and WHO-5. The MI group only showed an effect in the outcome measure Empowerment Scale (*d* = 0.85) after 30 sessions. In contrast, the LI group experienced a small effect (*d* = 0.47) after 15 sessions, which remained the same after 30 sessions (*d* = 0.43). A similar pattern appeared in the reduction of psychosocial health symptoms after 30 sessions in the MI group (HEALTH-49, *d* = −1.81), whilst the LI group showed a medium effect after 15 sessions (*d* = −0.72) and a large effect after 30 sessions (*d* = −1.35). Notably, the LI group showed a considerable increase in wellbeing after 15 sessions (WHO-5, *d* = 2.67) and 30 sessions (*d* = 6.06), three times more than the MI group (*d* = 2.32). Personality disorder severity (SASPD) did not change in the MI group. The LI group experienced a negligible effect on the reduction of severity (SASPD, *d* = −0.31) after 30 sessions. In summary, the quantitative data reveals that both groups experienced enhancements in treatment outcomes. This improvement is evidenced by increased levels of empowerment and wellbeing and a decrease in psychosomatic health complaints throughout 30 sessions, with both groups displaying similar patterns of progress.

To check whether Gestalt therapy was conducted in the sessions, the psychotherapist self-reported on the GTFS (Supplementary Figure S1) after each session. Results showed that Gestalt concepts in the therapy sessions were predominantly applied. Working with embodied awareness and experimental attitude was slightly less reported in sessions. To develop an understanding of the client's therapy processes over 30 sessions in both groups, we analyzed quantitative measures from the clients' therapy diaries. Descriptive data from clients' therapy diaries (Table 1) show clients' average experiences during the sessions—higher numbers indicate higher agreement with the dimension. The LI group revealed lower ratings in all subscales, especially in “body awareness” and “dialogical relational” compared to the MI group, which we further investigated in the qualitative diary entries.

**Table 1:** Clients' therapy diary.

**Clients' therapy diaries**	**Total (*****N** = * **135)**		**MI (*****N** = * **73)**		**LI (*****N** = * **63)**	
	***M***	***SD***	***M***	***SD***	***M***	***SD***
Intensity of process	4.09	0.96	4.50	0.75	3.68	1.16
Expressing a concern	4.33	0.89	4.60	0.66	4.07	1.12
Openness to experiments	3.93	1.20	4.42	1.04	3.44	1.36
Body awareness	3.47	1.20	4.11	1.07	2.82	1.33
Emotional awareness	4.36	0.80	4.53	0.71	4.19	0.88
Dialogic relational	3.76	0.95	4.28	0.77	3.25	1.14

Mean values of the self-report questionnaire in clients' therapy diaries during 30 sessions of Gestalt therapy. MI, moderately integrated personality structure; LI, low integrated personality structure; *M*, mean; *SD*, standard deviation.

### 3.2 Analysis of qualitative data

To explore the psychotherapy process, we analyzed external, internal, and reflective narrative sequences and the relational focus of sequences of all cases (RQ2) in both the therapist's and the client's diaries. Using the NPCS (Hardtke et al., 2002), we identified types of narrative sequences, relationship foci, and domain and facet shifts in the psychotherapist's diaries (*N* = 192 entries, comprising 85 MI and 107 LI) analyzing within- and across-group analysis as well as co-occurrences of frequencies.

It is important to note that we applied the same coding framework for “narrative sequences,” “relationship foci,” “domain shifts,” and “facet shifts” in both the therapist's and clients' diaries. To showcase examples of our coding of “narrative sequences” (“external,” “internal,” and “reflexive”) and “relational foci” (“others,” “self in relation to others,” and “self”), we will first refer to quotations from the psychotherapist's diary entries. Subsequently, we will refer to the client's diary entries to illustrate our coding of “domain shifts” and “facet shifts.” All example quotes have been translated from German to English.

Entries in the psychotherapist's diaries include all therapy sessions and give a good overview of the clients' therapy processes, as well as reporting interventions from the psychotherapist. For example, the psychotherapist described the following NPCS in the therapy diary: (a) external NPCS recounting events reported by the client (e.g., “the client spoke about a recent argument with a colleague at work”), (b) internal NPCS delving into the client's subjective or experienced emotions and reactions related to the events (e.g., “the client expressed feeling hurt and undervalued during that argument”), and (c) reflexive NPCS pertaining to the client's analytical reflections on these events, encompassing cognitive and emotional aspects (e.g., “the client analyzed the argument, realizing a pattern of defensiveness stemming from past experiences”). In NPCS, the coding of a process sequence is intertwined with a focus on relationships. When emphasizing “others,” the quotation pertains to people beyond the client. Placing the “self in relation to others” signifies examining the client's own position in connection to others. Conversely, discussing the “self” involves reflections on the client's own situation. Below, we present excerpts from the psychotherapist's diary, highlighting coded quotations with different relationship foci:

“The client reports stories about her ancestors from the maternal and paternal sides. She is interested in the family's history and would like to learn more about it” (LI client, external NPCS, relationship focus: others).

“The client is angry about her friend, who has reduced contact since the COVID-19 pandemic. The client feels alone, experiencing that she has to do everything herself and would like to be seen and supported by others” (LI client, internal NPCS, relationship focus: self in relation to others).

“The client reduces contact with her parents but is repeatedly affected and hurt by her father's comments pushing beyond her limits. [...] In the experiment [symbolizing with objects], it turns out that her father exerts control and power and has an illusory view of the world where everything is good, which does not reflect the real situation of the family […] that resulted in confusion in her childhood, as she experienced a discrepancy she could not deal with. The client realized that she desires to be seen, also in the therapeutic relationship between us” (MI client, reflexive NPCS, including an experience-oriented intervention, relationship focus: self).

Client diaries lack the comprehensiveness of psychotherapists' diaries, with some entries missing altogether. The length of the entries varied between clients from half to one full page of text. Notably, the MI group wrote four times more words in their therapy diary, and entries were more consistent over the 30 sessions.

In the following, we use quotations from clients to illustrate our coding of “domain shifts” and “facet shifts.” “Domain shifts” indicate changes in topics, while “facet shifts” reveal additional facets of the same topic. For instance, clients documented in their therapy diaries:

“We talked about my father and the situation with him at the moment [mental illness]. About how I don't want to talk to him at the moment, but at the same time feel guilty about not calling him. We also talked about being an adult and how good it is for me to be independent now and how great it is to be able to make my own decisions [in daily life]” (LI client, domain shift).

In this example, the client first talks about the situation with her mentally ill father and subsequently transitions to a different topic—making independent decisions as an adult in daily life situations, separate from her mother and brother, indicating a domain shift. In the subsequent example, the client discusses the bodily reactions experienced when feeling overwhelmed and establishes a connection to a subsequent action in response to those physical sensations, specifically setting boundaries, indicating a facet shift.

“It's actually great that my body shows me when it's enough for me [overwhelming situations] and I'm allowed to act accordingly - that I'm entitled to draw boundaries when I feel them” (MI client, facet shift).

To further explore how clients perceived their therapy process (RQ3), we analyzed positive experiences both within and outside therapy sessions and emotional reactions in the sessions reported in clients' therapy diaries (*N* = 135 entries, thereof 62 MI and 73 LI). For example, one client reported a “positive experience” made outside therapy, detailing an “emotional expression” of feeling proud during a challenging conversation with her mother in the therapy diary:

“I realized in that situation [talking to mother] that I was becoming defiant, but I told her [mother] clearly how much this statement affected me. I was proud then and I still am now. […] I have already learned a lot, namely, to stand by my feelings and to express them” (client 3, MI).

To examine which factors led to a successful therapy outcome empowering the client (RQ4), we analyzed four qualitative semi-structured interviews conducted with two representatives of the MI group and two from the LI group. For analyzing the interviews, we used the coding system developed from the diaries, supplemented with inductive categories. We quantified all codes as described above.

#### 3.2.1 Characteristics and experiences of the “moderately integrated” group

**Figure 4:**
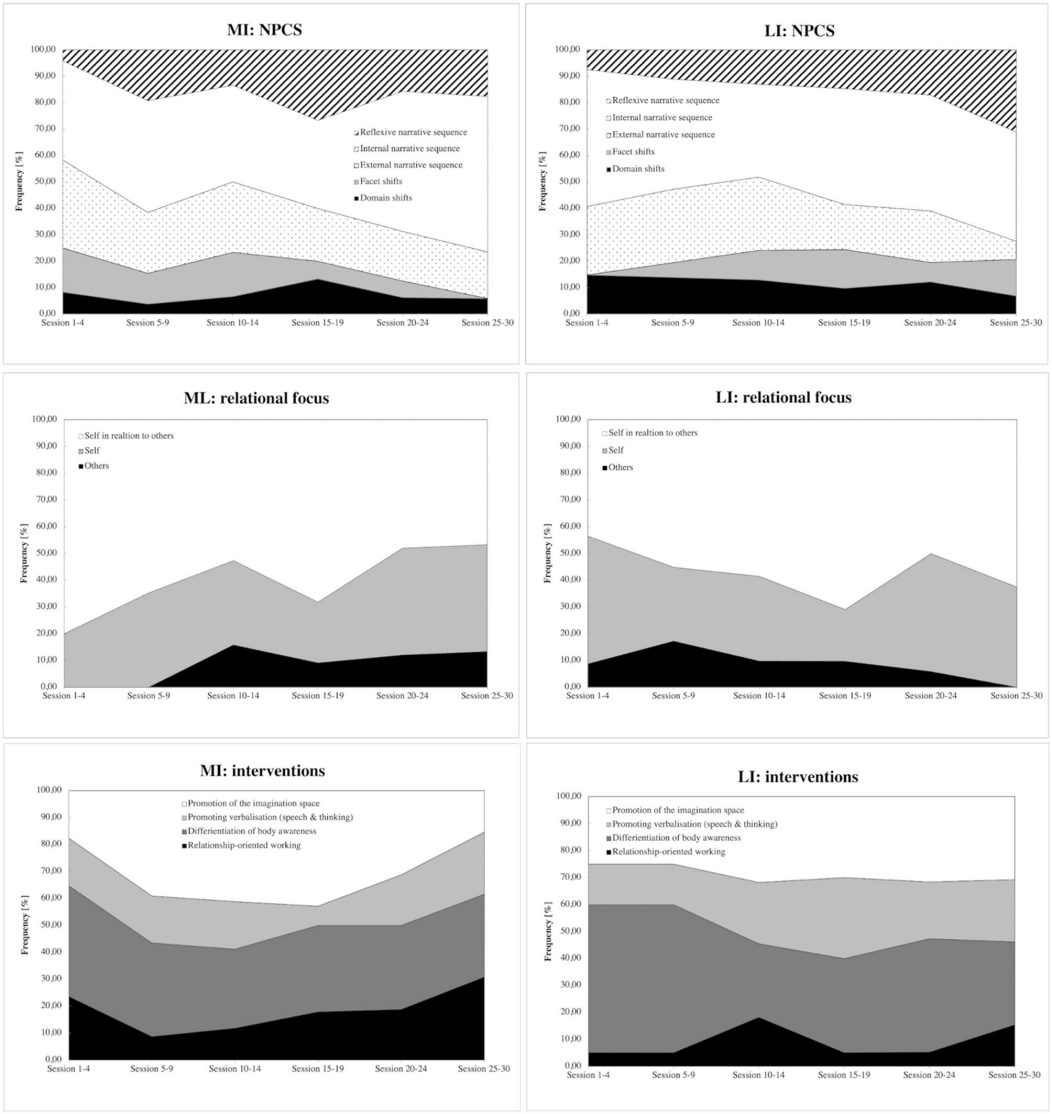
Narrative process coding system (NPCS). The figure shows narrative process coding sequences, relationship focus, and interventions documented over 30 therapy sessions in the moderately integrated (MI) and low-integrated (LI) groups.

Characteristics of the psychotherapy process of the MI group (Figure 4, Supplementary Table S5) showed that external narrative coding sequences in the psychotherapist's diary decreased from the first to the last session. In contrast, internal narrative coding sequences and reflexive narrative coding sequences increased over time. Clients talked more than half of the time about themselves in relation to others, one-third of the time about themselves, and less often about others. Similarly, more than half of the time, psychotherapist's interventions addressed the enhancement and differentiation of body awareness and promoting the imagination space, such as working with symbolisation, dreams, and self-aspects through experiments and distancing techniques from traumatic events in internal and reflexive narrative sequences. The psychotherapist also focused on relationship-orientated working, such as self-revelations, clarifications regarding relationships, sharing resonance, and adapting and tuning the level of difficulties for experiments in internal narrative sequences. This increased toward the last sessions.

In the interviews at the end of the study, MI clients reported four empowering factors in the therapy process: therapy supports self-regulation, offers relationships, promotes self-efficacy, and promotes experience (Supplementary Table S6). They felt empowered in the areas of resources and competencies, self-awareness, and self-efficacy. The major factor of empowerment addressed in this group is referred to “therapy supports self-regulation.” For example, a client reported how she managed intrapsychic regulation of emotions by staying calm and sorting her feelings in a difficult conversation with her parents:

“And so it [the atmosphere] was partially charged by my parents, but I remained quite matter-of-fact and tried to calm them down. When I noticed that they were getting louder or more emotional, I tried to counteract them somehow, and I probably wouldn't have been able to do that so well without the therapy. And in the meantime, I can sort out my feelings successfully” (client 2, MI).

MI clients reported in their therapy diaries that they had positive experiences in therapy, including a change of perspectives, the recognition of their own progress, and self-regulation in their therapy diary (Supplementary Table S7). Outside therapy, clients experienced overcoming old behavior patterns, setting active boundaries, and recognizing their own progress, as positive experiences. Most MI clients succeeded in transferring these insights from psychotherapy into the real world, especially drawing on their own resources and competencies, being open to new things, and regarding social support. Our subsequent anchoring example shows intrapsychic emotional regulation and awareness of needs; the client reported her inner process of allowing unpleasant feelings and emotions without displacing them through distraction with music or podcasts.

“[…] and now I've been trying for a bit longer to at least give in to these maybe not-so-nice feelings and not to push them away somehow immediately, but just to accept them and maybe think about them and not immediately distract myself with music or podcasts or whatever but just think about them”(client 3, MI).

**Figure 5:**
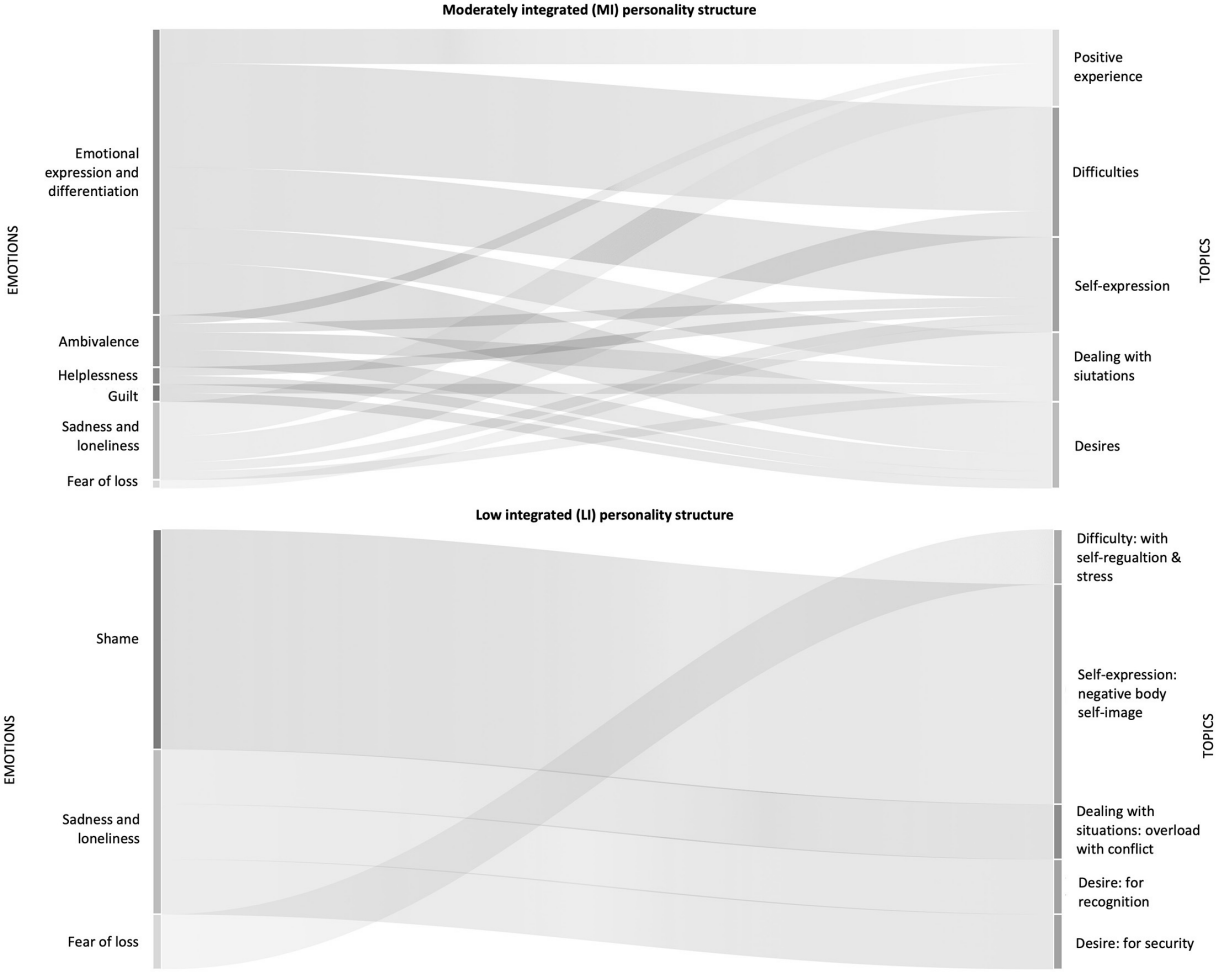
Emotional expressions co-occur with topics. On the left side, emotional expressions are listed that occur together with different topics (on the right side) displayed in a Sankey graph for the moderately integrated (MI) and low-integrated (LI) groups.

One-third of MI clients' diary entries addressed emotions describing inner ambivalence, chaos and helplessness, and self-expressions of longing, gratitude, attention seeking, and mortification. In almost all diary descriptions of difficulties, desires, self-expression, and dealing with situations, the clients used emotional expressions to describe their experiences (Supplementary Table S8). Figure 5 shows the co-occurrence of emotional descriptions with topics represented in the therapy diaries. For example, sadness is associated with a positive experience with self-regulation and emotional expression, and differentiation connects with difficulties in perceiving and admitting emotions. Guilt is matched with desires to address and express things to others.

The following MI client therapy diary text examples illustrate the attempt to explain certain emotions and feelings, making meaning, and finding explanations. The client shared her introspective exploration of trying to understand how family dynamics impact other relationships in her life. Recognizing the family dynamic enables her to derive meaning from her emotions of feeling small and her behavior with friends:

“I often feel like I am running away from myself and [avoid] standing up for myself. […] [I avoid accepting] that loneliness is good for me because it shows me what I long for. It is up to me to be more courageous and get what I want. It [new understanding of family dynamics] encourages me to try new things, leave old ways, and break out of myself. From family structures, from friendships, from my habits that keep me small, from everything that does not serve me and keeps me small” (client 2, MI).

#### 3.2.2 Characteristics and experiences of the “low-integrated” group

According to the psychotherapist's diary, the characteristics of the psychotherapy process in the LI group included the finding that external narrative coding sequences decreased from the first to the last session (Figure 4, Supplementary Table S5). In contrast, internal narrative coding sequences and reflexive narrative coding sequences increased. However, the LI group showed many “domain shifts” indicating that these clients shift topics more often within sessions and fewer “facet shifts”—exploring topics from different perspectives—in the first sessions. Furthermore, they talked more about themselves and others in the first sessions. The psychotherapist focused more on the differentiation of body awareness, imagination space, and promoting verbalisation (speech and thinking) as interventions in the first 10 sessions. Mainly, the differentiation of body awareness, such as awareness exercises, brain spotting, eye movement desensitization reprocessing (EMDR), and skill training, played a crucial intervention in all narrative coding sequences and promoting verbalisation, including metaphors, positive affirmations, and psychoeducation, especially in internal narrative coding sequences, where clients elaborate on experienced emotions and reactions to the events.

LI clients reported in the interviews that “therapy promotes experience,” “therapy offers relationship,” and “therapy promotes self-efficacy” are equally important for empowering clients in Gestalt treatment (Supplementary Table S6). Notably, therapy as support for self-regulation was mentioned less in this group. The following examples illustrate the promotion of experience through exercises, as a client reported feeling light and free after using the technique of brainspotting in the session, commenting in the therapy diary: “*I think it was a bit more than relaxation” (client 4, LI)*. In the subsequent example, a different client recounts her participation in an experiential exercise and the positive effects it had on her:

“[…] when X [the therapist], for example, said something yes, do we do that [experience-orientated exercise]? […] And then you go along with it. Whether it is now [in therapy] […] or outside [therapy], I once again tried something different. I notice that this [participating in this type of exercise] is simply extremely good for me”(client 5, LI).

Reporting of positive experiences in clients' therapy diaries included recognizing their own performances and progress, relaxing, and experiencing a sense of acceptance in therapy sessions (Supplementary Table S7). Outside therapy, most of the time, LI clients experience recognizing their own performance and progress, and relaxing and overcoming old behavior patterns as positive experiences. Notably, they also mentioned that “therapy does not help to expand the network of relationships.” This aspect was also seen in interviews in a lack of transfer of experience and knowledge gained in the therapy into the real-world setting, for example, focusing on interpersonal communication and the lack of developing self-assurance. A client experiencing challenges in interpersonal interactions described her difficulty in reaching out to others, anticipating unfavorable reactions from them:

“I've never done that before [organizing a meeting], and I managed to write to people and not be upset or anything because I was just afraid of that, and I managed very well. […] I was afraid of the appointment because maybe all the unpleasant people will get in touch or maybe, I don't know, 29 [people] would write back: ‘Thanks, but I'm not interested', but I was able to motivate a few people to go” (client 1, LI).

Only a few LI clients' diary entries dealt with difficulties, desires, and self-expressions. Half the entries described dealing with situations such as feeling overwhelmed with everyday life, relationships, and conflicts, and less on desires and difficulties (Supplementary Table S8). They mainly expressed negative emotions such as jealousy, shame, guilt, sadness, and fear of loss. Furthermore, self-expressions focused on negative body image, difficulties with self-responsibility and caring, and problems with self-esteem and self-worth. For example, shame is associated with the self-expression of a negative body self-image. Fear of loss is experienced together with difficulties with self-regulation and stress (Figure 5). Notably, positive experiences did not correlate with emotional experiences at all. The following client's therapy diary text examples illustrate the struggle to find explanations and make sense of emotions and bodily felt feelings. The client believes she is unattractive after enduring years of bullying related to her weight:

“Am I ugly, or do I just see it that way because I have experienced such things [bullying because of being overweight]? Of course, I could improve physically, etc. But I think the perception [of my body] I have right now stems from my experiences. It will be a long time until I accept myself as I am or actively find the motivation to deal with my body and sports” (client 5, LI).

### 3.3 Comparison of the MI and LI group

This comparison between groups considers the initial 30 sessions of Gestalt therapy treatment for seven female clients, and as such, its generalisability is limited. We examined the outcomes and psychotherapeutic processes of three cases from the moderately integrated group and four cases from the low-integrated group. In general, both groups exhibited comparable trends in treatment outcomes based on quantitative measures, demonstrating improvements in empowerment, wellbeing, and a decrease in psychosocial health complaints over the course of 30 sessions of Gestalt therapy. Notably, there were no discernible changes in the level of personality functioning during the treatment (Figure 3). However, there was a difference in how successful treatment outcomes were attained between the two groups, indicating distinct approaches in practice. These differences in the psychotherapeutic processes encompassed various aspects, including the focus on relationships and interventions (Figure 4) employed, factors contributing to empowerment, and the processing of emotions during therapy sessions (Figure 5), as described in the following.

First, over the course of 30 sessions, clients in both groups exhibited a similar distribution of external (description of events), internal (elaboration on experienced emotions and reactions related to the events), and reflexive narrative sequences (reflections on these events encompassing cognitive and emotional aspects), as analyzed from qualitative diary entries maintained by the psychotherapist (Figure 4). However, a distinction arose in terms of the relationship focus encoded in the narrative process sequences: The MI group tended to engage more in discussions about themselves in relation to others, indicative of a reflective process and perspective-taking. In contrast, during the initial 10 sessions, the LI group predominantly focused on themselves. Domain shifts addressed shifts to different topics within the narrative coding sequence, underscoring the challenge of directing attention inward rather than toward others. This might be associated with the establishment of a trustworthy therapeutic alliance, as reflected in the therapy diaries' first section (including a 5-point Likert scale), where the LI group rated “dialogical relational” lower than the MI group at the outset of treatment (Table 1).

Second, the psychotherapist employed experience-oriented interventions (Figure 4), particularly emphasizing body awareness, and encouraged creativity through imagination in both groups. This emphasis was particularly noticeable in the internal process sequences that delved into emotions and bodily sensations, as discerned from the psychotherapist's therapy diaries. However, the differentiation of body awareness was more frequently utilized in all narrative process coding sequences within the LI group. Notably, the LI group also demonstrated a focus on verbalisation in internal process sequences. The process of verbalisation facilitated the expression of feelings and bodily sensations, aiding in awareness and the discovery of meaning in emotional processes. This emphasis on verbalisation is also reflected in the quantitative data from clients' therapy diaries, as evidenced by lower ratings in the LI group on the dimensions of “emotional- and body- awareness” (Table 1).

Third, the post-treatment client interviews revealed distinct factors contributing to empowerment in the two groups (Supplementary Table S6). The MI group found that “therapy supports self-regulation,” enabling them to manage emotions intrapsychically, a notion mirrored in positive experiences within and outside therapy. Conversely, the LI group highlighted the importance of “therapy promotes experience,” “offers relationship,” and “promotes self-efficacy” as equally crucial for empowerment in Gestalt therapy—factors they associated with the therapy session and the therapeutic relationship. This experience underscored the significance of co-regulation, being able to relax, feel accepted in therapy, and recognize personal progress both within and outside therapy sessions. However, the LI group did not connect these experiences with positive emotions, potentially explaining the lack of improvement in empowerment as an outcome measure in the latter half of therapy (Figure 3). Only the MI group–possibly due to successful self-regulation processes, which are linked to emotional processing—demonstrated the ability to translate these experiences into real-world scenarios and manifested an increase in empowerment according to the outcome measure.

Finally, as previously noted, emotional expressions and processing differ in both groups (Figure 5, Supplementary Table S7), as evidenced by clients' therapy diaries. MI individuals undergo a spectrum of both positive and negative emotions, detailed in emotional and self-expressions within the therapy diaries. In contrast, the LI group predominantly encounters negative emotions that overshadow their self-expression, such as feelings of shame and a negative body image (Supplementary Table S8). Interestingly, the LI group did not report positive emotions linked to positive experiences, even though such experiences are acknowledged both within and outside therapy. This suggests challenges in emotional processing and the interpretation of emotions.

## 4 Discussion

In this multiple case study comparing two groups, we explored the treatment effects of Gestalt therapy in clients with common mental health disorders and structural problems in a naturalistic individual psychotherapy setting. We assessed treatment outcomes with standardized questionnaires at three time points and process outcomes with therapy diaries and semi-structured interviews. We compared cases with “moderately integrated” (MI) or “low-integrated” (LI) personality structures according to OPD (2014) on an outcome and process level.

### 4.1 Treatment outcome: quantitative measures

The comparison of cases of quantitative measures in the MI and LI groups revealed similar patterns in both groups regarding successful treatment outcomes (Figure 3, Supplementary Table S4). Both the MI and LI groups showed an increase in empowerment (Empowerment Scale), wellbeing (WHO-5), and a reduction of psychosocial health complaints (HEALTH-49) after 15 and 30 sessions that align with previous outcome research in Gestalt therapy (Schigl, 1998; Bargghaan et al., 2002; Harfst et al., 2003; Elliott et al., 2004, 2021; Struempfel, 2006a; Hartmann-Kottek, 2014). Changes in the level of personality functioning (SASPD) did not occur during treatment. However, we observed a reduction of symptoms at the beginning of therapy that can be explained with the three-phase model of psychotherapy outcome (Howard et al., 1993). This model entails sequential improvements of subjectively experienced wellbeing with its mobilization of hope, followed by a reduction in symptomatology and enhancement in life functioning. However, to enable empowerment, clients must reflect on their situation (Kliche and Kroeger, 2008) to find, articulate, and realize their interests (Knuf, 2004; Prins, 2007; Reichhart et al., 2008). This might develop as part of life functioning and self-development later in therapy and depends on the availability of secondary ego-functions, such as empathy and metallization. The latter fosters slow change processes of personality affecting life functioning. Moreover, the OPD (2014) describes the personality structures as “not rigid and unchanging but shows lifelong development processes […] here is the point of contact with concepts such as identity, character or personality … a slow change model” (p. 114). This view is in accordance with Gestalt theory, describing self-development as dynamic and fluid (Perls et al., 2015; Staemmler, 2015; Spagnuolo-Lobb, 2016b); a process that requires a dialogical development in contact with others (Stern, 1985; Wheeler, 2000; Spagnuolo-Lobb, 2012; Staemmler, 2015, 2018; Buber, 2017; Boeckh, 2019a).

### 4.2 Therapy process: qualitative measures

The LI group generally wrote less in their therapy diaries than the MI group, with a ratio of 1:4. This could be attributed to their reflecting functions, considering their personality functioning structure and higher stress levels, as they expressed a need for relaxation and support with self-regulation during sessions—factors the MI group found empowering. Additionally, the LI group may have had less interest or time for reflection.

We first normalized codes in therapy diaries using Atlas.ti software to avoid bias resulting from unequal therapy diary lengths, allowing for relative frequency-based group comparisons rather than absolute counts. This approach is useful when documents vary in length or document groups have different sizes, preventing misleading comparisons based on absolute frequencies (Friese, 2021). This normalization enabled us to compare cases at a group level rather than individual cases. Second, we analyzed the psychotherapist's diaries (not the clients') to assess processes. This was because the therapists' entries were more consistent in both length and content, offering a detailed view of the clients' journey across 30 sessions.

While the MI group reported self-regulation as a major empowerment factor in the post-treatment interview, the LI group emphasized that therapy promotes experience, relationships, and self-efficacy (Supplementary Table S6). For example, an MI client reported self-regulating aspects in the therapy diary by staying calm and sorting her feelings in a difficult conversation with her parents. In contrast, a LI client reported in the therapy diary that participation in an experiential exercise had positive effects on her, emphasizing the significance of the experiential aspect of the exercise. From a Gestalt therapy perspective, people with low-integrated personality structure lack the certainty of being existent, and primary ego-functions are not sufficiently available, such as perceiving and feeling (Hochgerner and Schwarzmann, 2018). However, these functions can be promoted in experience-oriented exercises in sessions, such as working with imagination, creative techniques, and body awareness exercises. Especially, emotion- and experience-activating Gestalt dialogues achieve greater awareness of implicit feelings and convictions in the therapeutic relationship to access childhood memories, phantasies, and feelings (Struempfel, 2006a,b).

The LI group also experienced more interventions related to the body and imagination, which boosted their verbalisation (Supplementary Table S5), as reported in the psychotherapist's diaries. This emphasis on verbalisation was evident in the LI group's internal processes. Similar patterns were observed in Gestalt therapy for psychosomatic patients with varying degrees of integrated personality structures. This underscores the value of verbalizing therapeutic processes to solidify lasting therapeutic impacts. A focus on body awareness not only enhances self-awareness but also aids in understanding, feeling, and communication. Such techniques can enhance emotional and bodily consciousness by promoting reflective processes (Hochgerner, 2015). This supports personal growth (Petzold, 1993) and the internalization of relational experiences (Rudolf et al., 2008), thereby evoking greater depths of experience and emotional activation (Greenberg et al., 2003; Greenberg, 2015). The integration of new experiences may be accelerated by deeply felt bodily sensations, which give rise to new understandings through intense in-session emotional moments (Samoilov and Goldfried, 2000; Greenberg, 2002). This process enhances the capacity to manage self-esteem and emotions, allowing for a clearer distinction between experiences and feelings. It aids in assimilating perceptions of others and one's self-image, as well as internalizing relationships (Rudolf et al., 2008).

Another explanation relates to the contact between the client and therapist that forms the foundation for emotional regulation, the further ability to mentalize, and the integration of ego-functions (Wöller, 2013). Studies showed that a solid therapeutic alliance is a prerequisite for processing emotions (Beutler et al., 2000; Iwakabe et al., 2000; Horvath, 2005). This builds on Perls et al. (2015) contact-support model that helps develop contact functions through enough support, which emphasizes working with support in treatment (Votsmeier, 1999; Votsmeier-Röhr, 2005, 2011). From a psychodynamic view, experiences need to be emotionally evaluated and carried together in the situation to learn verbal differentiation of emotions and create the experience of the body-self (Rudolf, 2013). If successful, the self can regulate self-image and self-worth and has the ability to control and act (OPD, 2014). These functions rely on inner images for self- and affect-regulation (Fonagy et al., 2002), which the MI group described as a significant factor in therapy, namely, self-regulation.

From a Gestalt therapy view, the personality structure is “a set of psychic functions and their internal cohesion, which allows the person to self-regulate and creatively adapt in the organization of his life and to find identity and self-worth” (Votsmeier, 1999, p. 715). Clients with limitations in contact and relationship functions often rely heavily on the therapist to act as a direct and proactive partner (Hochgerner et al., 2018). This is because they are overwhelmed by emotions and struggle to connect with internalized relationships or inner perceptions of objects (OPD, 2014). In brief, during the early years of life, an individual's sense of self is shaped and nurtured through interpersonal interactions. These interactions lead to the creation of memories from lived experiences (Stern, 2011), which in turn shape one's perception of self, others, and relationship dynamics, often associated with specific emotions (Kernberg, 1981; Bacal et al., 1994; Sandler and Sandler, 1999). Emotional expression plays a pivotal role in this, either by reaching out to another person or being emotionally impacted by them (Rudolf, 2013).

This perspective underscores the importance of dialogical engagement in Gestalt therapy (Wheeler, 2000; Spagnuolo-Lobb, 2012; Perls et al., 2015; Buber, 2017). It also highlights the need for therapists to adopt a supportive approach, especially when assisting individuals with structural deficits. Clients with low integration often feel physical tension and a sense of detachment, symptoms of desensitization, and limited bodily sensations. These symptoms can be interpreted as coping mechanisms stemming from overstimulation or developmental challenges (Petzold, 1993), which might be reflected in the low scores on body and emotional awareness scales (Table 1). Supporting this, experiential therapy research (Greenberg and Pascual-Leone, 1995; Greenberg, 2002) suggests that merely being aware of emotions is not enough for meaningful change. Actual change requires merging thought and emotion, aligning with the reflective and mentalizing functions outlined in OPD (2014). Building on these findings, we further argue that bodily felt experiences charged with emotional experiences are necessary to deeply process emotions in terms of re-evaluation and integration of experiences.

Positive experiences reported in the MI clients' therapy diaries were associated with emotional and self-expressions (Supplementary Table S6). For example, an MI client described a positive experience with intrapersonal emotion regulation as an inner process of allowing unpleasant feelings and emotions before displacing them through distraction, i.e., music or podcasts. In contrast, a LI client facing difficulties in interpersonal interactions articulated the challenge of reaching out to others and managing social interactions. While recounting a positive experience, her narrative underscored a lack of self-assurance, evident in her anticipation of unfavorable reactions from colleagues.

These examples underline aspects of structural functioning, namely, requiring differentiation of self- and object-relation, the ability to regulate emotions and self-worth, and the ability to be emphatic and mentalize (OPD, 2014). These reflecting functions modulate self- and emotion-regulation (Fonagy et al., 2002). On the other hand, the LI group displayed negative emotions such as shame, guilt, sadness, and fear of loss. These emotions were linked to poor body image and challenges with self-esteem (Supplementary Table S7, Figure 5). Emotional expressions did not co-occur with positive experiences in this group. This could be attributed to constraints in the profound processing of emotions, which are essential for the development of a self-image connected to inner objects, as discussed in Rudolf (2002, 2013) and OPD (2014). Moreover, these findings suggest a restriction in reflecting upon positive emotions and the process of deriving meaningful insights from these emotions, which is essential for long-lasting transformation (Greenberg and Pascual-Leone, 1995; Greenberg, 2002), involving the utilization of bodily sensations to construct fresh interpretations (Petzold, 1977, 1993; Samoilov and Goldfried, 2000; Greenberg, 2002).

Previous research on Gestalt therapy treatment according to structural deficits (Hochgerner and Schwarzmann, 2018) reported that working with body awareness releases tension and fosters relaxation. However, verbalizing feelings and experiences (reflecting process) has only short-term therapeutic effects on low-integrated personality structure. Another aspect of lacking emotional processing could be the quality of the therapeutic alliance since emotional arousal is mediated by a strong alliance predicting good outcomes (Beutler et al., 2000; Horvath, 2005) and awareness of inner bodily feelings (Gendlin, 1996).

It is worth noting that this study spans only the initial year of Gestalt therapy. This duration might be restrictive, as changes in personality structures are a slow change process (OPD, 2014). More extended periods might be necessary to fully integrate positive experiences and interactions, leading to more balanced self-regulation (Perls et al., 2015). Finally, the study's findings indicate an initial boost in wellbeing and a reduction in psychosomatic symptoms. It should be noted that these improvements do not necessarily translate to long-term positive outcomes in severe personality disorders (Howard et al., 1993).

### 4.3 Limitations and strengths

Our study's findings have specific boundaries when considering their applicability to broader contexts. The sample, treatment type, and the cultural and societal backdrop of the study all influence its transferability. The study exclusively involved female clients undergoing Gestalt therapy, limiting its generalisability to other demographics or therapeutic approaches. The small participant count further narrows the scope of our interpretations. Additionally, the study involved a single trainee psychotherapist familiar with OPD (2014). However, we believe the psychotherapist maintained consistency in their approach and techniques throughout.

The study was conducted in a naturalistic environment devoid of randomization and standardized guidelines. This approach facilitated a genuine mix of clients with varied personality structures and individualized treatments. It is worth noting that we applied the NPCS (Hardtke et al., 2002) to diary reflections and semi-structured interviews, even though it was originally designed for analyzing transcribed therapy session scripts. Additionally, we did not utilize clinically validated structured or standardized interviews to evaluate the clients' personality structures, such as the Structured Interview of Personality Organization (STIPO-R, Clarkin et al., 2016) or OPD-Structure Questionnaire (OPD-SF, Schauenburg et al., 2012).

While our study primarily focused on group-level comparisons, individual nuances were factored into the therapy diary analysis by normalizing the codes. This allowed for group comparisons using the Atlas.ti software. A more in-depth exploration of individual variations is planned for a subsequent publication. Finally, while the quantitative results indicated effective Gestalt treatment, the strong bond between the therapist and clients by the study's conclusion might have influenced these outcomes. Whilst there is a possibility that clients felt inclined to give positive feedback in the standardized questionnaires, inflating the reported benefits, our data do not substantiate this theory, as we observed no change in personality functioning (SASPD) during or post-treatment.

The extent to which our findings can be generalized is further constrained by the fact that the comparison between the MI and LI groups encompasses only the initial 30 sessions of Gestalt therapy treatment for three moderately integrated and four low integrated female clients. Nevertheless, the existing empirical data on structural functioning and Gestalt treatment (Hochgerner and Schwarzmann, 2018) align with our findings. Additionally, our data align cohesively with Gestalt theory (e.g., Perls et al., 2015; Staemmler, 2015; Buber, 2017) and psychodynamic developmental theories (e.g., Rudolf, 2013; OPD, 2014). Therefore, we posit that these results carry implications for practical application.

A significant strength of our study lies in its mixed-method design, which allowed for data triangulation and comparison. We achieved a comprehensive dataset by combining quantitative outcome measures with diverse qualitative data sources such as therapy diaries and interviews. Although we lacked real-time session data, such as audio recordings, the post-session reflections provided by clients and the psychotherapist offer valuable insights. These reflections likely capture pivotal moments and realizations, even if they do not encompass every detail documented in therapy diaries.

## 5 Conclusion

This study utilized a mixed-methods approach to compare two groups in a multiple-case setup: individuals with moderately integrated (MI) and low-integrated (LI) personality structures. In over 30 sessions of Gestalt therapy, the study delved into empowerment and self-development. Both groups exhibited positive outcomes in empowerment, wellbeing, and psychosocial health. However, they differed in their therapeutic journey, interventions, and empowerment factors leading to successful outcomes. Notably, no changes were observed in personality functioning levels. Future research should look deeper into the lasting effects of therapy across both groups, aiming to understand better how to support personality functioning during therapy. This includes focusing on embodied emotions in Gestalt therapy with a more extensive clinical sample. It would benefit subsequent studies to incorporate standardized OPD assessments, especially personality structure-related ones. Additionally, other clinically relevant tests focusing on emotion, body, and mentalizing should be considered to comprehensively understand the mechanisms in personality functioning that influence self-development and growth in Gestalt therapy.

Given the outlined limitations, there are several practical implications for Gestalt therapy in the initial 30 sessions of treatment. For clients with low-integrated personality structures seeking empowerment and positive outcomes, co-regulating during therapy sessions is essential, offering enriching experiences to enhance self-efficacy. A primary focus should be placed on interventions centered on body awareness, with a gradual shift toward verbalizing emotional and physical sensations. It is also beneficial to introduce relaxation techniques, encouraging clients to recognize their progress and changes in perspective and fostering a sense of acceptance. Furthermore, therapists should aim to connect positive experiences, both within and outside the therapy environment, with emotional and bodily sensations.

On the other hand, for clients with moderately integrated personality structures, the goal remains empowerment and positive outcomes. A foundational step is to establish a supportive therapeutic relationship, placing emphasis on self-efficacy in areas such as resources, competencies, self-awareness, decision-making, and autonomy. This foundation aids in enhancing empowerment. Additionally, therapists should guide clients in exploring topics from diverse perspectives, drawing attention to old behavioral patterns. Such an approach is instrumental in helping clients gain deeper insights and drive positive changes throughout their therapy journey.

## Data availability statement

The original contributions presented in the study are included in the article/Supplementary material, further inquiries can be directed to the corresponding author.

## Ethics statement

The studies involving humans were approved by Ethics Commission University of Continuing Education Krems Donau-University Krems, Dr.-Karl-Dorrek-Straße 30, 3500 Krems Austria. The studies were conducted in accordance with the local legislation and institutional requirements. The participants provided their written informed consent to participate in this study. Written informed consent was obtained from the individual(s) for the publication of any potentially identifiable images or data included in this article.

## Author contributions

RK: Conceptualization, Formal analysis, Methodology, Project administration, Visualization, Writing – original draft, Writing – review & editing. MF: Formal analysis, Writing – review & editing. UD: Conceptualization, Supervision, Writing – review & editing. TP: Conceptualization, Methodology, Supervision, Writing – review & editing. YS: Conceptualization, Formal analysis, Methodology, Supervision, Writing – review & editing, Writing – original draft.
