# Supplementary material for: Common mental disorders in Gestalt therapy treatment: a multiple case study comparing patients with moderate and low integrated personality structures
Source: Front Psychol. 2023 Dec 20;14:1304726. doi: 10.3389/fpsyg.2023.1304726 (PMC10761483; doi:10.3389/fpsyg.2023.1304726)
Supplement: Supplementary file 1 [file Data_Sheet_1.pdf]

Supplementary Material

Supplementary Table 1. Demographic data of the MI and LI group at treatment onset.

	**MI (*N* = 3)**		**LI (*N* = 4)**		**Total**	
	M	*SD*	*M*	*SD*	*M*	*SD*
Age	28.22	3.06	33.40	12.84	30.79	9.48
Gestalt therapy sessions	28.33	2.89	26.80	3.95	27.54	3.42
ICD-10 diagnoses codes	F32.1. F33.1. F41.2		F32.1. F43.1. F43.1. F60.3		-	
Demographic data	Count	%	Count	%	Count	%
Education						
Secondary schools incl. college	0	0.00	3	42.86	3	42.86
University. academy	3	42.86	1	14.29	4	57.14
Employment						
Employee	2	28.57	3	42.86	5	71.43
Student	1	14.29	1	14.29	2	28.57
Marital status						
Single	2	28.57	3	42.86	5	71.43
Married. (un)registered partnership	1	14.29	1	14.29	2	28.57
Prior experience with psychotherapy						
Yes (few months to up to 4 years)	1	14.29	2	28.57	3	42.86
No	2	28.57	2	28.57	4	57.14
Currently in psychiatric care						
Yes	1	14.29	2	28.57	3	42.86
No	2	28.57	2	28.57	4	57.14
Intake of psychopharmaceutic						
Yes	0	0.00	1	14.29	1	14.29
No	3	42.86	3	42.86	6	85.71

Note. MI = moderately integrated personality structure. LI = low integrated personality structure. % = absolute frequencies in percent. *M* = mean. *SD* = standard deviation.

Supplementary Figure 1. Gestalt Therapy Fidelity Scale. Overview of psychotherapist's application of Gestalt concepts in all therapy sessions (*N* = 192).

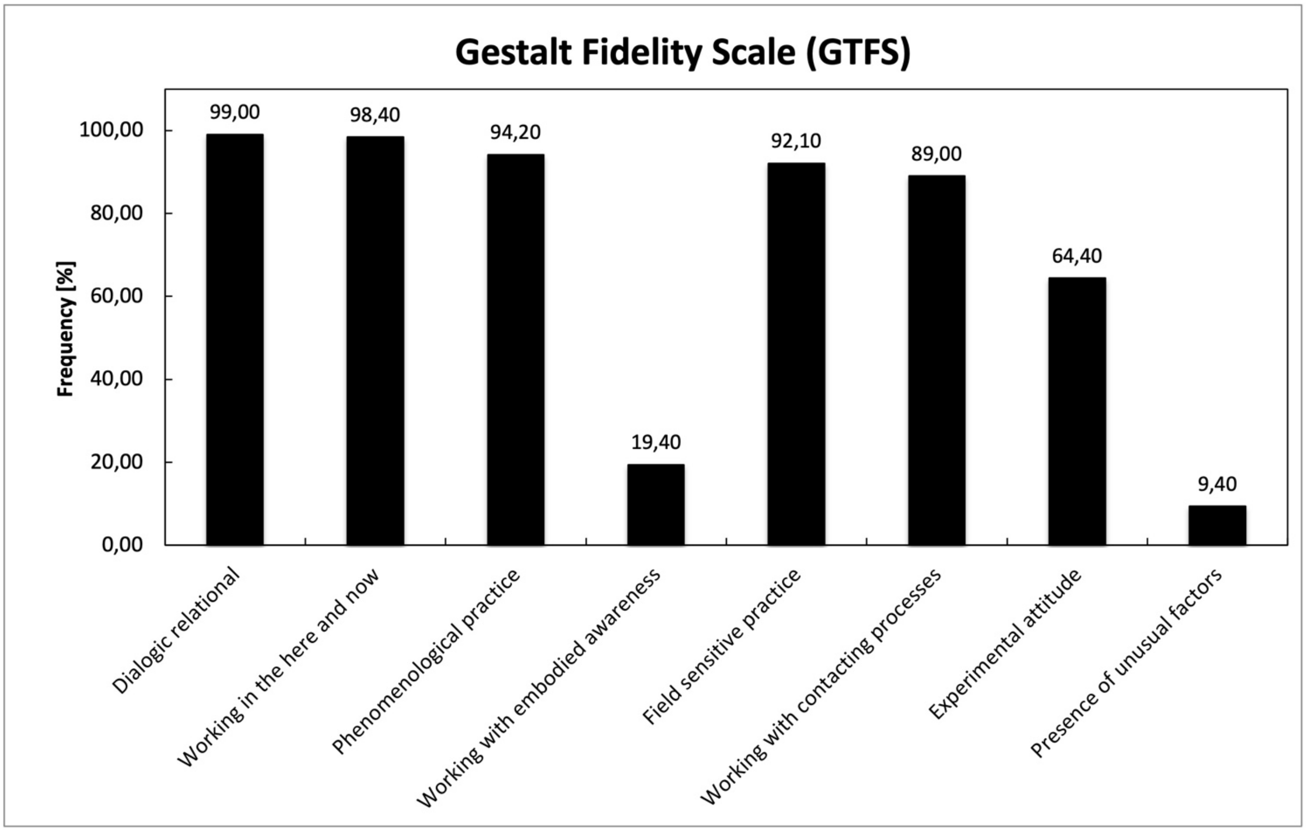

Supplementary Table 2. Semi-structured interview guideline.

Theme	Question
Self-awareness and self-efficacy	Please tell me about a difficult situation in the last few weeks (i.e. since the beginning of therapy). how were you able to overcome this situation on your own?
	Please tell me about a situation within the last few weeks where you felt you could make a difference. How does that feel to you on a physical level? When you feel this way now. do you also feel what emotions are involved?
Decision-making and autonomy	Please remember a situation within the last few weeks. were there situations where you felt like you had to make decisions yourself or be involved in decision making? Can you tell me about it?
	Please tell me about a situation within the last few weeks where you had to make an important decision. Was anything different here. compared to previous situations? Can you tell me what was different here?
Resources and competences	Please tell me about a situation within the last few weeks in which you strengthened or rediscovered your resources and skills.
	Were there also situations outside of therapy in which you were able to apply these skills? Can you tell me about them?
Social support	Please tell me about a situation in which you felt supported by people (friends, family, etc.) in recent weeks. What did these people do differently compared to before? How did you notice this?
Capacity for change and openness	Please tell me about a situation within the last few weeks where you feel something has changed in your interactions with others?
	What was their contribution to this change (for example, their behavior, attitude, experience, etc.)? Can you tell me about it?
	Please tell me about a situation within the last few weeks where you were open to or engaged with something new?
Debrief	Is something missing? Is there anything else you would like to tell?

Supplementary Table 3. Coding list. Categories and (sub)codes used for coding of diaries and interviews.

Source	Category	Subcategory	Codes
Therapy diaries of psychotherapist	Therapy sessions	1-14 sessions	1-4 sessions, 5-9 sessions, 10-14 sessions
		15-30 sessions	15-19 sessions, 20-24 sessions, 25-30 sessions
	Narrative process coding system (NPCS)	External process coding sequence	
		Internal process coding sequence	
		Reflexive process coding sequence	
		Domain shifts	
		Facet shifts	
		Relationship focus	Self, others, self in relation to others
	Interventions	Relationship-oriented working	Self-revelation, clarifications in the relationship, sharing resonance, adapting and tuning the level of difficulties for experiments with the client
		Differentiation of body awareness	Body awareness exercises, brain spotting, eye movement desensitisation reprocessing (EMDR), skill training, symbolisation with material
		Promoting verbalization (speech & thinking)	Metaphors, positive affirmations, psychoeducation
		Promotion of the imagination space	Scenic work, working with self-aspects, working with dreams, hot seat, thought experiments, distancing techniques from traumatic events, imaginations
Therapy diaries of clients	Topics	Difficulties	Perceiving/met needs. maintaining own boundaries, perceiving/admitting emotions, closeness and distance to others, self-care, self-regulation and stress, self-responsibility
		Desires (for)	To be seen, recognition, security, control, new relationship, rest and relaxation, change, to belong, to address/express things, different approach to therapy
		Emotions	(Emotional) expressiveness, jealousy, ambivalence, helplessness, powerlessness, emotional chaos, shame, guilt, feeling of security, sadness and loneliness, abandonment / fear of loss
		Self-expressions	(Self-)reflection, attention, gratitude, identity, mortification, negative beliefs, negative body self-image, longing, self-deprecation and self-doubt, self-evaluation, self-responsibility and caring, self-esteem / self-worth
		Dealing with situations	Detachment from family, expectations and hopes, pressure to perform, men in relationships, traumatic experience, overload in everyday life, assaultive behavior, own performance, conflict, doubts about the relationship

	Positive experiences	In psychotherapy session	
		Outside psychotherapy session	
		Actively set boundaries	
		Overcome old behaviour patterns	
		Recognise own performance and progress	
		Relaxation	
		Sense of acceptance	
		Change of perspective	
		Self-empowerment	
		Self-regulation	
Interviews with clients	Themes	Self-awareness and self-efficacy	
		Resources and competences	
		Social support	
		Capacity for change and openness	
		Decision-making and autonomy	
	Specific factors in therapy	Therapy offers relationship	Therapeutic relationship builds trust and openness, therapy offers an exclusive relationship, therapy can take a long time
		Therapy promotes experiences	Therapy enables a change of perspective, therapy helps prepare for challenges, therapy works through the use of exercises
		Therapy promotes self-efficacy	Therapy enables (self-) confrontation, therapy enables (further) development, therapy helps to discover resources and competences, therapy is help for self-help
		Therapy promotes self-regulation	Therapy helps regulate emotions, therapy creates bodily references, therapy supports setting boundaries
		Therapy does not help to expand the network of relationships	
	Transfer into real-world setting		

S4 Table. Outcome measures at three time points. Mean scale values of psychometric questionnaires at baseline. 15 sessions and 30 sessions psychotherapy.

	Baseline						15 sessions psychotherapy								
	Total		MI		LI		Total			MI			LI		
	M	*SD*	*M*	*SD*	*M*	*SD*	*M*	*SD*	*d*	*M*	*SD*	*d*	*M*	*SD*	*d*
Empowerment Scale (Rogers et al. 1997)	*3.35*	*0.47*	*3.46*	*0.39*	*3.23*	*0.55*	*3.51*	*0.43*	*0.35*	*3.53*	*0.18*	*0.17*	*3.49*	*0.67*	*0.47*
Self-esteem. self-efficacy	3.38	0.73	3.44	0.38	3.31	1.07	3.56	0.64	0.26	3.63	0.13	0.48	3.50	1.16	0.18
Power	2.95	0.79	3.08	0.90	2.81	0.68	3.09	0.57	0.18	3.13	0.25	0.05	3.06	0.88	0.37
Community activism and autonomy	4.31	0.14	4.44	0.10	4.17	0.19	4.36	0.25	0.38	4.39	0.10	-0.58	4.33	0.41	0.87
Optimism and control over the future	3.41	0.40	3.75	0.25	3.06	0.55	3.19	0.25	-0.54	3.25	0.25	-2.00	3.13	0.25	0.11
Righteous anger	2.70	0.79	2.58	0.58	2.81	1.01	3.34	0.99	0.81	3.25	0.90	1.15	3.44	1.09	0.62
Severity of personality disorder (SASPD)	*0.86*	*0.39*	*0.74*	*0.28*	*0.97*	*0.51*	*0.83*	*0.22*	*-0.06*	*0.78*	*0.11*	*0.13*	*0.89*	*0.33*	*-0.16*
Psychosocial health (HEALTH-49)	*2.53*	*0.52*	*2.33*	*0.53*	*2.74*	*0.50*	*2.24*	*0.54*	*-0.57*	*2.11*	*0.36*	*-0.43*	*2.38*	*0.72*	*-0.72*
Psychosomatic complaints (PSB)	2.08	0.35	1.89	0.36	2.28	0.34	1.93	0.56	-0.45	1.69	0.34	-0.56	2.17	0.78	-0.33
Somatoform complaints (SOM)	1.89	0.51	1.86	0.25	1.93	0.78	1.68	0.66	-0.41	1.48	0.50	-1.54	1.89	0.82	-0.05
Depression (DEP)	2.85	0.49	2.33	0.73	3.38	0.25	2.33	0.62	-1.08	1.94	0.19	-0.54	2.71	1.04	-2.67
Phobic anxiety (PHO)	1.29	0.20	1.33	0.31	1.25	0.10	1.63	0.76	1.64	1.60	0.72	0.87	1.65	0.79	4.00
Psychological Wellbeing (WOHL)	3.31	0.30	2.67	0.42	3.95	0.19	2.68	0.60	-2.06	2.27	0.46	-0.96	3.10	0.74	-4.44
Interactional problems (INT)	2.92	0.72	2.76	1.00	3.07	0.44	2.48	0.50	-0.61	2.67	0.30	-0.09	2.29	0.70	-1.77
Self-efficacy (SELB)	3.03	0.48	2.60	0.40	3.45	0.55	2.40	0.34	-1.31	2.40	0.20	-0.50	2.40	0.49	-1.91
Activity and participation (A&P)	2.86	1.01	2.72	1.06	3.00	0.95	2.53	0.36	-0.32	1.94	0.35	-0.73	3.13	0.37	0.13
Social support (SOZU)	2.44	0.61	2.50	0.50	2.38	0.72	2.34	0.51	-0.15	2.50	0.25	0.00	2.19	0.77	-0.26
Social stress (SOZB)	2.68	0.49	2.67	0.29	2.69	0.69	2.42	0.51	-0.53	2.58	0.29	-0.29	2.25	0.74	-0.64
Well-being (WHO-5)	*9.63*	*3.04*	*13.00*	*4.58*	*6.25*	*1.50*	*13.13*	*4.98*	*1.15*	*16.00*	*4.00*	*0.65*	*10.25*	*5.97*	*2.67*

	30 sessions psychotherapy								
	Total			MI			LI		
	M	*SD*	*d*	*M*	*SD*	*d*	*M*	*SD*	*d*
Empowerment Scale (Rogers et al. 1997)	*3.63*	*0.43*	*0.61*	*3.80*	*0.24*	*0.85*	*3.47*	*0.62*	*0.43*
Self-esteem. self-efficacy	4.07	0.63	0.95	4.33	0.29	2.31	3.81	0.96	0.47
Power	3.85	1.22	1.14	3.21	0.07	0.14	4.50	2.36	2.48
Community activism and autonomy	4.35	0.36	0.29	4.44	0.19	0.00	4.25	0.52	0.43
Optimism and control over the future	3.43	0.65	0.05	3.75	0.66	0.00	3.10	0.63	0.07
Righteous anger	3.13	0.56	0.54	3.25	0.25	1.15	3.00	0.87	0.19
Severity of personality disorder (SASPD)	*0.78*	*0.42*	*-0.20*	*0.78*	*0.22*	*0.13*	*0.78*	*0.62*	*-0.38*
Psychosocial health (HEALTH-49)	*1.72*	*0.64*	*-1.57*	*1.75*	*0.48*	*-1.81*	*2.08*	*0.80*	*-1.31*
Psychosomatic complaints (PSB)	1.52	0.44	-1.60	1.37	0.22	-1.53	1.71	0.65	-1.69
Somatoform complaints (SOM)	1.49	0.35	-0.79	1.33	0.36	-0.99	1.36	0.34	-0.73
Depression (DEP)	1.76	0.84	-2.25	1.61	0.48	-1.65	2.38	1.19	-3.98
Phobic anxiety (PHO)	1.63	0.37	1.64	1.13	0.23	2.18	1.25	0.50	0.00
Psychological Wellbeing (WOHL)	2.30	0.65	-3.32	2.00	0.00	-1.60	2.60	1.30	-7.05
Interactional problems (INT)	2.08	0.94	-1.16	1.76	0.64	-1.00	2.39	1.24	-1.53
Self-efficacy (SELB)	2.16	0.80	-1.82	2.07	0.69	-1.33	2.25	0.90	-2.18
Activity and participation (A&P)	1.88	0.84	-0.97	1.72	0.90	-0.94	2.04	0.77	-1.01
Social support (SOZU)	2.16	0.39	-0.46	2.00	0.25	-1.00	2.31	0.52	-0.09
Social stress (SOZB)	2.50	0.81	-0.36	2.50	1.00	-0.58	2.50	0.61	-0.27
Well-being (WHO-5)	*15.75*	*4.30*	*2.01*	*18.00*	*1.00*	*1.09*	*13.50*	*7.60*	*4.83*

Supplementary Table 5. Therapy process of MI and LI group. Within-group comparisons of baseline. 15 sessions. and 30 sessions of psychotherapy.

Within-group comparison: MI	Total duration (30 session)		Session 1-14 (N=3)		Session 15-30 (N=3)		Session 1-4 (N=3)		Session 5-9 (N=3)		Session 10-14 (N=3)		Session 15-19 (N=3)		Session 20-24 (N=3)		Session 25-30 (N=2)	
**Diary psychotherapist**	**Count**	**%**	**Count**	**%**	**Count**	**%**	**Count**	**%**	**Count**	**%**	**Count**	**%**	**Count**	**%**	**Count**	**%**	**Count**	**%**
Narrative sequences	*154*	*100,00*	*80*	*100,00*	*74*	*100,00*	24	*100,00*	26	*100,00*	30	*100,00*	30	*100,00*	32	*100,00*	17	*100,00*
External narrative sequence	37	24,03	22	27,50	15	20,27	8	33,33	6	23,08	8	26,67	6	20,00	6	18,75	3	17,65
Internal narrative sequence	65	42,21	31	38,75	34	45,95	9	37,50	11	42,31	11	36,67	10	33,33	17	53,13	10	58,82
Reflexive narrative sequence	25	16,23	10	12,50	15	20,27	1	4,17	5	19,23	4	13,33	8	26,67	5	15,63	3	17,65
Domain shifts	12	7,79	5	6,25	7	9,46	2	8,33	1	3,85	2	6,67	4	13,33	2	6,25	1	5,88
Facets shifts	15	9,74	12	15,00	3	4,05	4	16,67	3	11,54	5	16,67	2	6,67	2	6,25	0	0,00
Relational focus	*109*	*100,00*	*51*	*100,00*	*58*	*100,00*	*15*	*100,00*	*17*	*100,00*	*19*	*100,00*	*22*	*100,00*	*25*	*100,00*	*15*	*100,00*
Others	10	9,17	3	5,88	7	12,07	0	0,00	0	0,00	3	15,79	2	9,09	*3*	12,00	*2*	13,33
Self	33	30,28	15	29,41	18	31,03	3	20,00	6	35,29	6	31,58	5	22,73	*10*	40,00	*6*	40,00
Self in realtion to others	66	60,55	33	64,71	33	56,90	12	80,00	11	64,71	10	52,63	15	68,18	*12*	48,00	*7*	46,67
Intervention	*113*	*100,00*	*57*	*100,00*	*56*	*100,00*	*17*	*100,00*	*23*	*100,00*	*17*	*100,00*	*28*	*100,00*	*16*	*100,00*	*13*	*100,00*
Relationship-oriented working	19	16,81	8	14,04	11	19,64	4	23,53	2	8,70	2	11,76	5	17,86	3	18,75	4	30,77
Differientiation of body awareness	38	33,63	20	35,09	18	32,14	7	41,18	8	34,78	5	29,41	9	32,14	5	31,25	4	30,77
Promoting verbalisation (speech & thinking	18	15,93	10	17,54	8	14,29	3	17,65	4	17,39	3	17,65	2	7,14	3	18,75	3	23,08
Promotion of the imagination space	38	33,63	19	33,33	19	33,93	3	17,65	9	39,13	7	41,18	12	42,86	5	31,25	2	15,38
Positive experiences	*18*	*100,00*	*6*	*100,00*	*12*	*100,00*	*1*	*100,00*	*2*	*100,00*	*3*	*100,00*	*5*	*100,00*	*7*	*100,00*	*3*	*100,00*
in psychotherapy sessions	14	77,78	*4*	66,67	*10*	83,33	*0*	0,00	*0*	0,00	*2*	66,67	*5*	100,00	*5*	71,43	*2*	66,67
outside pschotherapy sessions	4	22,22	*2*	33,33	*2*	16,67	*1*	100,00	*2*	100,00	*1*	33,33	*0*	0,00	*2*	28,57	*1*	33,33

Note. MI = moderately integrated personality structure, % = absolute frequency in percent.

Within-group comparison: LI	Total duration (30 session)		Session 1-14 (N=4)		Session 15-30 (N=4)		Session 1-4 (N=4)		Session 5-9 (N=4)		Session 10-14 (N=4)		Session 15-19 (N=4)		Session 20-24 (N=4)		Session 25-30 (N=3)	
**Diary psychotherapist**	**Count**	**%**	**Count**	**%**	**Count**	**%**	**Count**	**%**	**Count**	**%**	**Count**	**%**	**Count**	**%**	**Count**	**%**	**Count**	**%**
Narrative sequences	*207*	*100,00*	*124*	*100,00*	*83*	*100,00*	27	*100,00*	36	*100,00*	54	*100,00*	41	*100,00*	41	*100,00*	29	*100,00*
External narrative sequence	48	23,19	33	26,61	15	18,07	7	25,93	10	27,78	15	27,78	7	17,07	8	19,51	2	6,90
Internal narrative sequence	87	42,03	51	41,13	36	43,37	14	51,85	15	41,67	19	35,19	18	43,90	18	43,90	12	41,38
Reflexive narrative sequence	30	14,49	15	12,10	15	18,07	2	7,41	4	11,11	7	12,96	6	14,63	7	17,07	9	31,03
Domain shifts	26	12,56	17	13,71	9	10,84	4	14,81	5	13,89	7	12,96	4	9,76	5	12,20	2	6,90
Facets shifts	16	7,73	8	6,45	8	9,64	0	0,00	2	5,56	6	11,11	6	14,63	3	7,32	4	13,79
Relational focus	*166*	*100,00*	*99*	*100,00*	*67*	*100,00*	*23*	*100,00*	*29*	*100,00*	*41*	*100,00*	*31*	*100,00*	*34*	*100,00*	*24*	*100,00*
Others	16	9,64	11	11,11	5	7,46	2	8,70	5	17,24	4	9,76	3	9,68	2	5,88	0	0,00
Self	54	32,53	33	33,33	21	31,34	11	47,83	8	27,59	13	31,71	6	19,35	15	44,12	9	37,50
Self in realtion to others	96	57,83	55	55,56	41	61,19	10	43,48	16	55,17	24	58,54	22	70,97	17	50,00	15	62,50
Intervention	*107*	*100,00*	*66*	*100,00*	*41*	*100,00*	*20*	*100,00*	*20*	*100,00*	*22*	*100,00*	*20*	*100,00*	*19*	*100,00*	*13*	*100,00*
Relationship-oriented working	10	9,35	7	10,61	3	7,32	1	5,00	1	5,00	4	18,18	1	5,00	1	5,26	2	15,38
Differientiation of body awareness	38	35,51	22	33,33	16	39,02	11	55,00	11	55,00	6	27,27	7	35,00	8	42,11	4	30,77
Promoting verbalisation (speech & thinking	26	24,30	15	22,73	11	26,83	3	15,00	3	15,00	5	22,73	6	30,00	4	21,05	3	23,08
Promotion of the imagination space	33	30,84	22	33,33	11	26,83	5	25,00	5	25,00	7	31,82	6	30,00	6	31,58	4	30,77
Positive experiences	*17*	*100,00*	*6*	*100,00*	*11*	*100,00*	*0*	*0,00*	*4*	*100,00*	*2*	*100,00*	*3*	*100,00*	*8*	*100,00*	*5*	*100,00*
in psychpotherapy	12	70,59	*4*	66,67	*8*	72,73	*0*	0,00	*3*	75,00	*1*	50,00	*2*	66,67	*5*	62,50	*3*	60,00
outside pschotherapy	5	29,41	*2*	33,33	*3*	27,27	*0*	0,00	*1*	25,00	*1*	50,00	*1*	33,33	*3*	37,50	*2*	40,00

Note. LI = low integrated personality structure, % = absolute frequency in percent.

Supplementary Table 6. Specific factors of empowerment in Gestalt therapy. Across-group and within-group comparisons between the MI and LI group displayed in relative frequencies.

	Across-group				Within-group	
	MI		LI		MI	LI
Specific factors of empowerment	Count	%	Count	%	%	%
Therapy offers relationship	*4.00*	*34.97*	*7.44*	*65.03*	*6.45*	*12.00*
(Therapeutic) relationship creates trust & openness	4.00	61.73	2.48	38.27	6.45	4.00
Therapy offers exclusive relationship	0.00	0.00	2.48	100.00	0.00	4.00
Therapy can take a long time	0.00	0.00	2.48	100.00	0.00	4.00
Therapy promotes experience	*2.00*	*21.19*	*7.44*	*78.81*	*3.23*	*12.00*
Therapy enables a change of perspective	2.00	100.00	0.00	0.00	3.23	0.00
Therapy helps prepare for challenges	0.00	0.00	2.48	100.00	0.00	4.00
Therapy works using exercises	0.00	0.00	4.96	100.00	0.00	8.00
Therapy promotes self-efficacy	*4.00*	*34.97*	*7.44*	*65.00*	*6.45*	*12.00*
Therapy enables (self-)confrontation	2.00	100.00	0.00	0.00	3.23	0.00
Therapy enables (further) development & growth	1.00	100.00	0.00	0.00	1.61	0.00
Therapy helps to discover resources and competences	0.00	0.00	7.44	100.00	0.00	12.00
Therapy is help for self-help	1.00	100.00	0.00	0.00	1.61	0.00
Therapy supports self-regulation	*4.00*	*61.73*	*2.48*	*38.27*	*6.45*	*4.00*
Therapy helps to regulate emotions	1.00	100.00	0.00	0.00	1.61	0.00
Therapy creates bodily references	3.00	100.00	0.00	0.00	4.84	0.00
Therapy supports setting boundaries	0.00	0.00	2.48	100.00	0.00	4.00
Therapy does not help to expand the network of relationships	*0.00*	*0.00*	*2.48*	*100.00*	*0.00*	*4.00*
Transfer to real-world setting	*34.00*	*77.41*	*9.92*	*22.59*	*54.84*	*16.00*

Note. All data points are normalised across cases and code frequencies. MI = moderately integrated personality structure. LI = low integrated personality structure. % = relative frequencies in per cent (total number of coding of all selected codes in the table).

Supplement Table 7. Positive experiences of clients in therapy. Across-group comparison of moderately integrated (MI) and low integrated (LI) groups regarding their positive experience during the psychotherapy process reported in clients' therapy diaries.

Across-group comparison of clients' positive experiences in therapy	Client's view			
	MI ($N = 3$)		LI ($N = 4$)	
	Count	%	Count	%
Positive experiences	49		49	
outside psychotherapy session	9.03	9.81	13.00	14.13
in psychotherapy session	39.97	38.45	36.00	34.62
Positive experience topics	48		48	
Actively set boundaries	4.27	21.40	1.00	5.02
Overcome old behaviour patterns	7.47	32.75	4.00	17.54
Recognise own performance and progress	7.47	21.73	14.00	40.75
Relaxation	1.07	5.38	9.00	45.35
Sense of acceptance	4.27	19.26	7.00	31.59
Change of perspective	7.47	65.12	4.00	34.88
Self-empowerment	6.40	23.45	4.00	14.66
Self-regulation	9.60	28.11	5.00	14.64

Note. All data points are normalised across cases and code frequencies. MI = moderately integrated personality structure. LI = low integrated personality structure. % = relative frequencies.

Supplementary Table 8. Clients' topics in therapy. Across-group comparison of topics addressed in the clients' therapy diaries.

	MI (*N* = 3)		LI (*N* = 4)	
Topics	Count	%	Count	%
Difficulties	*35.00*	*16.55*	*28.43*	*13.45*
Difficulty in perceiving/met needs	7.00	15.68	7.11	15.92
Difficulties in maintaining own boundaries	10.00	28.96	4.74	13.73
Difficulty in perceiving/admitting emotions	14.00	28.04	7.11	14.24
Difficulties with closeness and distance to others	14.00	28.04	7.11	14.24
Difficulties with self-care	1.00	4.02	2.37	9.53
Difficulties with self-regulation and stress	1.00	4.23	2.37	10.03
Difficulties with self-responsibility	5.00	10.33	7.11	14.68
Desires	*36.00*	*22.35*	*30.80*	*19.12*
Desire to be seen	3.00	7.07	0.00	0.00
Desire for recognition	3.00	21.24	4.74	33.54
Desire for security	4.00	12.59	2.37	7.46
Desire for control	3.00	100.00	0.00	0.00
Desire for new relationship	0.00	0.00	9.48	51.62
Desire for rest and relaxation	3.00	12.09	11.85	47.73
Desire for change	6.00	29.73	0.00	0.00
Desire to belong	5.00	41.09	0.00	0.00
Desire to address/express things	10.00	100.00	0.00	0.00
Desire for a different approach to therapy	2.00	45.77	2.37	54.23
Emotions	*52.00*	*33.91*	*37.91*	*24.73*
(Emotional) expressiveness	23.00	100.00	0.00	0.00
Ambivalence	7.00	100.00	0.00	0.00
Jealousy	2.00	45.77	2.37	54.23
Helplessness. powerlessness	2.00	100.00	0.00	0.00
Emotional chaos	4.00	100.00	0.00	0.00
Shame	1.00	7.14	4.74	33.86
Guilt	4.00	14.16	7.11	25.16
Feeling of security	1.00	4.77	2.37	11.30
Sadness and Loneliness	9.00	24.58	14.22	38.83
Abandonment / fear of loss	3.00	15.54	7.11	36.83
Self-expressions	*57.00*	*36.22*	*37.91*	*24.09*
(Self-)reflection	3.00	100.00	0.00	0.00
Attention	11.00	100.00	0.00	0.00
Gratitude	9.00	100.00	0.00	0.00
Identity	2.00	32.22	0.00	0.00
Mortification	5.00	100.00	0.00	0.00
Negative beliefs	5.00	27.64	0.00	0.00
Negative body self-image	0.00	0.00	4.74	33.64
Longing	2.00	100.00	0.00	0.00
Self-deprecation and self-doubt	12.00	28.23	9.48	22.29
Self-evaluation	2.00	20.04	2.37	23.74
Self-responsibility and caring	5.00	31.86	7.11	45.30

Self-esteem / self-worth	7.00	21.56	16.59	51.09
Dealing with situations	*51.00*	*21.03*	*101.89*	*42.02*
Detachment from family	11.00	20.70	16.59	31.21
Expectations and hopes	5.00	100.00	0.00	0.00
Pressure to perform	4.00	23.75	4.74	28.14
Men in relationships	0.00	0.00	14.22	54.23
Traumatic experience	0.00	0.00	2.37	12.55
Overload in everyday life	2.00	3.42	47.39	81.11
Assaultive behavior	3.00	28.12	2.37	22.21
Dealing with own performance	0.00	0.00	0.00	0.00
Dealing with conflict	15.00	31.80	16.59	35.16
Doubts about the relationship	13.00	100.00	0.00	0.00

Note. All data points are normalised across cases and codes frequencies. MI = moderately integrated personality structure. LI = low integrated personality structure. % = relative frequencies in percent (total number of coding of all selected codes in the table).
